# Regime-aware hybrid ensemble learning for adaptive $\hbox {PM}_{2.5}$ forecasting in urban environments

**DOI:** 10.1038/s41598-026-45437-w

**Published:** 2026-04-09

**Authors:** Juhi Kumari, Rajesh Wadhvani, Sanyam Shukla, Lalit Kumar

**Affiliations:** 1 Department of Computer Science and Engineering, Maulana Azad National Institute of Technology Bhopal, Bhopal, 462003 Madhya Pradesh India; 2 Department of Artificial Intelligence and Machine Learning, Manipal University Jaipur, Jaipur, 303007 Rajasthan India

**Keywords:** Air quality modeling, $\hbox {PM}_{2.5}$ forecasting, Atmospheric dispersion, CatBoost-TabNet model, Adaptive hybrid ensemble learning, Urban air pollution, Public health, Climate sciences, Environmental sciences, Mathematics and computing

## Abstract

Accurate forecasting of fine particulate matter PM2.5 is critical for mitigating adverse health and environmental impacts, yet existing models often fail to adapt to dynamic meteorological regimes. This study introduces a novel hybrid ensemble framework that adaptively switches between CatBoost and TabNet based on meteorological dispersion parameters, enabling context-specific model dominance. The approach was evaluated using the OpenAQ dataset, comprising hourly PM2.5 concentrations and corresponding meteorological variables across Delhi, India. The adaptive switching mechanism, driven by ventilation coefficient and relative humidity thresholds, resulted in CatBoost dominance for 58% of days, TabNet dominance for 35%, and balanced contributions for the remainder. Experimental results demonstrated that the proposed ensemble achieved an RMSE of 15.54, MAE of 11.02, MAPE of 12.2%, and $R^2$ of 0.86, outperforming established baselines such as LSTM, XGBoost, and LightGBM, with statistically significant improvements ($p<0.05$). Efficiency analysis revealed only a 14% increase in inference time compared to CatBoost alone, while offering substantial gains in predictive accuracy. Hyperparameter sensitivity analysis further identified an optimal configuration (learning rate = 0.01, batch size = 64) balancing convergence stability and generalization. By integrating model interpretability, adaptive learning, and computational efficiency, this research presents a scalable forecasting solution for urban air quality management. The methodology’s design allows seamless extension to other pollutants, geographic regions, and ensemble combinations, positioning it as a practical tool for data-driven environmental policy and public health protection.

## Introduction

**Figure 1:**
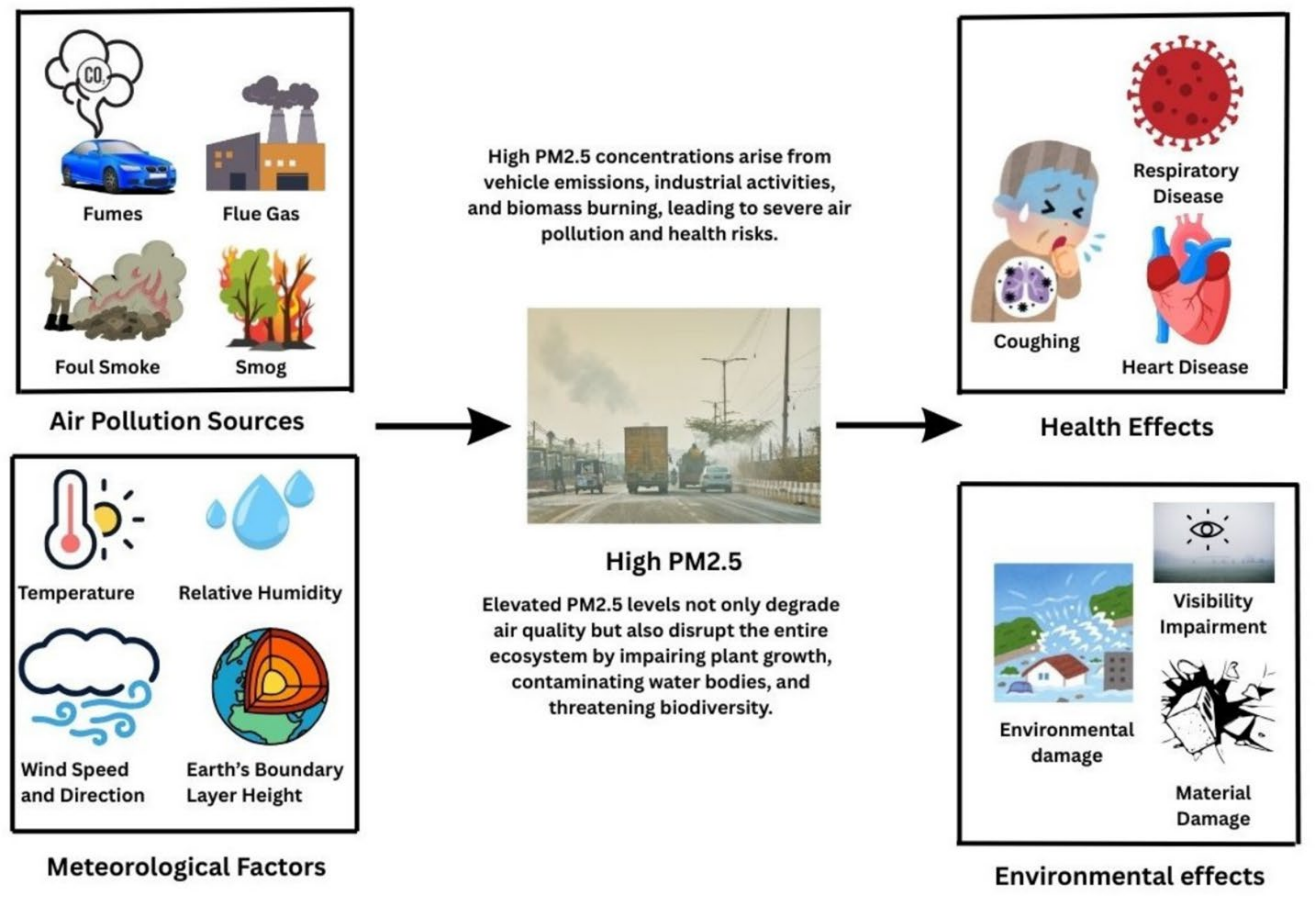
Schematic illustration of the major emission sources, meteorological factors, and their combined role in elevating $\hbox {PM}_{2.5}$ concentrations, along with the associated health and environmental impacts in urban regions.

Air pollution remains one of the most pressing environmental and public health challenges of the twenty-first century, contributing to an estimated 6.7 million premature deaths globally each year^1^. Among various pollutants, fine particulate matter with an aerodynamic diameter less than 2.5 micrometres ($\hbox {PM}_{2.5}$) poses the most severe health risks due to its ability to penetrate deep into the respiratory tract and enter the bloodstream^2^. Figure 1 shows causes and effects of high $\hbox {PM}_{2.5}$. Exposure to elevated $\hbox {PM}_{2.5}$ concentrations has been linked to cardiovascular diseases, respiratory illnesses, reduced cognitive function, and increased mortality^3^. In rapidly urbanising regions, this challenge is exacerbated by industrial growth, vehicular emissions, biomass burning, and unfavourable meteorological conditions that can trap pollutants over extended periods.

Delhi, the capital of India, has consistently ranked among the most polluted cities worldwide^4^. The city frequently experiences prolonged episodes of severe air quality, particularly during the post-monsoon and winter seasons, when agricultural stubble burning in neighbouring states coincides with low wind speeds, temperature inversions, and high relative humidity^5,6^. Studies have shown that annual average $\hbox {PM}_{2.5}$ levels in Delhi exceed the World Health Organization’s guideline values by more than ten times, placing millions of residents at continuous risk^1^. Furthermore, extreme pollution episodes have significant socio-economic impacts, leading to school closures, reduced workforce productivity, and increased healthcare burdens^7^. Beyond direct health impacts, poor air quality imposes significant economic costs through lost labour productivity, transportation disruptions, and heightened energy demand during pollution episodes. In India, policy measures such as the Graded Response Action Plan (GRAP) depend critically on the reliability of air quality forecasts, underscoring the need for models that are not only accurate but also robust across varying atmospheric regimes^8^.

To enable proactive mitigation actions, advise policymakers, and issue health advisories, accurate and timely $\hbox {PM}_{2.5}$ concentration forecasts is crucial. Although autoregressive integrated moving average (ARIMA) models and other traditional statistical techniques have been widely utilized for air quality forecasting, they are not very good at capturing non-linear connections between pollutant and meteorological factors^9^. By utilizing high-dimensional datasets and non-linear feature interactions, machine learning (ML) techniques such as gradient boosting, random forests, and deep learning architectures have demonstrated higher predictive performance^10^. However, three major challenges persist in the current literature. First, most models rely on static architectures that apply uniform model weighting across all forecast horizons and environmental conditions. This lack of adaptivity can limit performance under shifting pollution regimes, such as transitions between clean and polluted air masses, or between seasonal meteorological states. Second, despite the availability of high-quality meteorological data from reanalysis products such as ERA5 and real-time ground-based pollutant data from platforms like OpenAQ^11^, their combined utilisation in hybrid models remains limited. Multi-source data fusion has the potential to improve forecasting skill, yet integrating and downscaling such heterogeneous data remains technically challenging. Recent years have witnessed the rapid growth of open-access air quality and meteorological datasets, including reanalysis products such as ERA5, ground-based monitoring networks like CPCB and OpenAQ, and satellite-derived aerosol optical depth (MODIS, Sentinel). Low-cost sensor deployments have further improved spatiotemporal resolution across urban areas. While these advances offer unprecedented opportunities for high-resolution forecasting, challenges persist due to data heterogeneity, missing values, and scale mismatches, which complicate effective data fusion and model training. Third, there is a paucity of studies that explicitly evaluate model robustness under extreme pollution events, which are critical for public health response and mitigation planning. Furthermore, the interpretability of deep learning approaches remains limited, hindering their acceptance in policymaking contexts where transparent decision support is essential. Models trained for one urban environment also struggle to generalise across cities with distinct emission sources and meteorological regimes, raising concerns about spatial transferability. Finally, many frameworks underperform in forecasting rare yet extreme pollution episodes, which are precisely the events of greatest public health concern.

### Emerging machine learning approaches for forecasting

Forecasting environmental and climatic phenomena has increasingly relied on machine learning (ML) and deep learning (DL) models due to their capability to capture nonlinear relationships and complex spatiotemporal dependencies^12^. Earlier forecasting systems were mainly based on statistical techniques such as regression and autoregressive models, which often struggled to represent the dynamic behavior of environmental systems.

Recent studies have demonstrated the effectiveness of ML and DL techniques in various forecasting applications. Neural network based approaches have been applied for predicting carbon emissions and environmental pollution trends using large-scale temporal datasets^13,14^, providing useful insights for environmental monitoring and sustainability planning^15^. Similarly, hybrid deep learning frameworks integrating machine learning algorithms with neural networks have been successfully applied for air quality forecasting tasks such as $\hbox {PM}_{{10}}$ and $\hbox {PM}_{2.5}$ prediction, achieving improved predictive performance and interpretability using explainable AI techniques^16–18^.

Advanced deep learning architectures have also been explored for large-scale environmental forecasting^19^. Transformer-based models have demonstrated strong capability in capturing long-range temporal dependencies and spatial patterns for predicting global air pollution exposure trends^20^. In addition, physics-informed neural networks (PINNs) have been introduced to incorporate domain-specific physical constraints within the learning process, improving both predictive accuracy and physical consistency in meteorological forecasting tasks^21–24^. Hybrid and ensemble learning approaches have also gained significant attention for forecasting complex environmental systems. These frameworks combine the strengths of multiple machine learning and deep learning models to improve robustness and predictive accuracy. Recent studies demonstrate that ensemble frameworks integrating gradient boosting algorithms with neural networks can effectively capture nonlinear relationships in large environmental datasets^25^. Furthermore, hybrid architectures combining machine learning and deep learning techniques have shown promising results in solving complex scientific and engineering prediction problems^26,27^.

Despite these advancements, many forecasting frameworks rely on centralized datasets, which can introduce challenges related to data privacy, data sharing, and distributed data ownership. Federated learning (FL) has emerged as a promising paradigm to address these limitations by enabling collaborative model training across distributed data sources without transferring raw data. Additionally, recent research highlights the importance of AI-driven forecasting systems for climate-related challenges, including environmental monitoring and climate-driven disease prediction^28^.

### Motivation of the study and major contributions

Motivated by these developments, the present study proposes a federated ensemble weather forecasting framework that integrates transfer-learning-based models such as CatBoost and TabNet^29^. The proposed approach aims to leverage ensemble learning and federated optimization to improve forecasting accuracy while preserving data privacy across distributed meteorological datasets. Accurate and timely forecasting of $\hbox {PM}_{2.5}$ concentrations is essential for enabling proactive mitigation measures, informing policymakers, and issuing health advisories. Traditional statistical approaches, such as autoregressive integrated moving average (ARIMA) models, have been widely used for air quality forecasting but are limited in capturing non-linear relationships between meteorological and pollutant variables^30^. Machine learning (ML) methods, including gradient boosting, random forests, and deep learning architectures, have shown superior predictive performance by leveraging non-linear feature interactions and high-dimensional datasets^10^. However, three major challenges persist in the current literature.

First, most models rely on static architectures that apply uniform model weighting across all forecast horizons and environmental conditions. This lack of adaptivity can limit performance under shifting pollution regimes, such as transitions between clean and polluted air masses, or between seasonal meteorological states. Second, despite the availability of high-quality meteorological data from reanalysis products such as ERA5 and real-time ground-based pollutant data from platforms like OpenAQ, their combined utilisation in hybrid models remains limited. Multi-source data fusion has the potential to improve forecasting skill, yet integrating and downscaling such heterogeneous data remains technically challenging. Third, there is a paucity of studies that explicitly evaluate model robustness under extreme pollution events, which are critical for public health response and mitigation planning.

**Figure 2:**
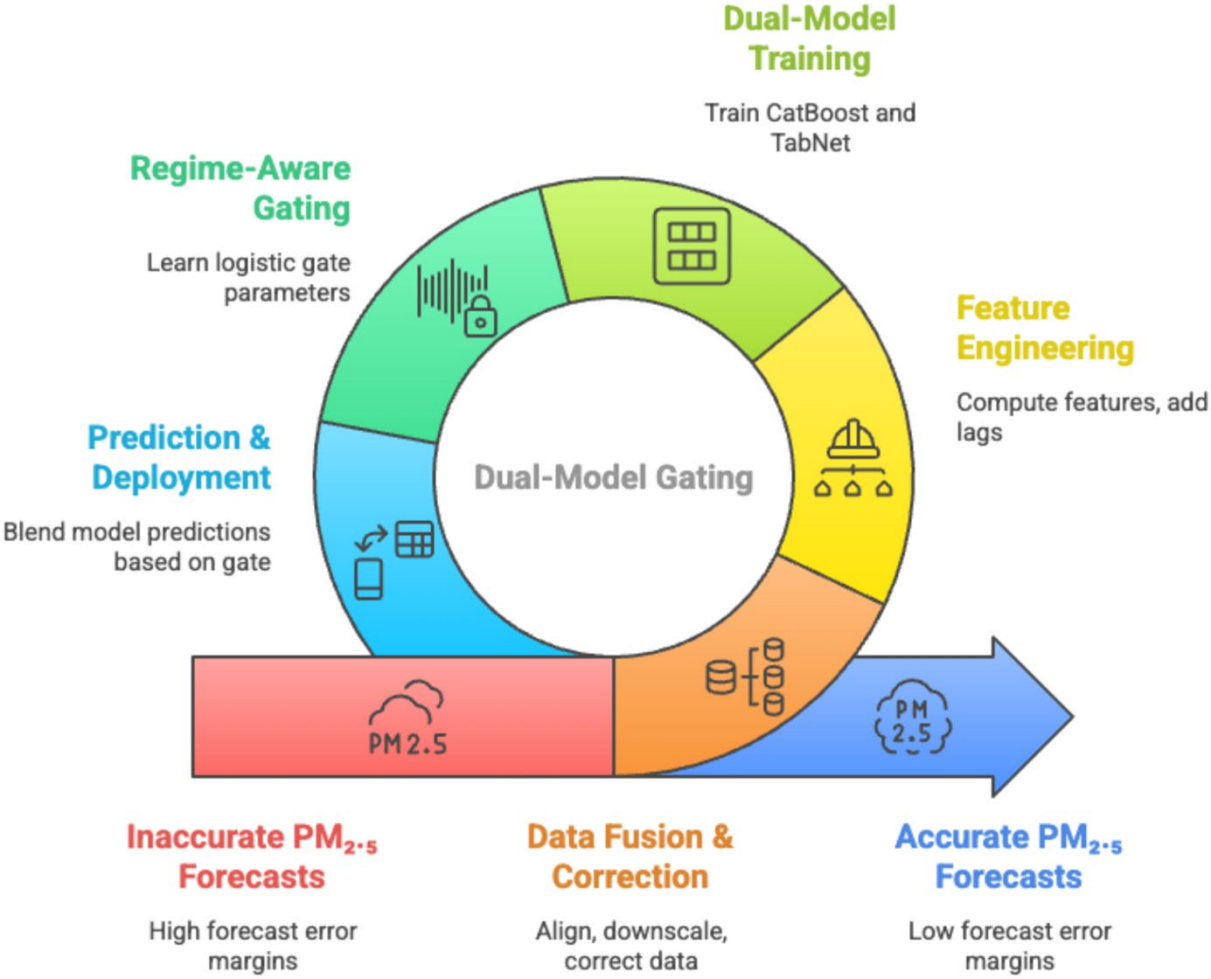
Proposed hybrid framework for feature downscaling and forecasting weather.

To address these gaps, this study proposes a regime-aware hybrid ensemble model as shown in Fig. 2 for daily $\hbox {PM}_{2.5}$ forecasting in Delhi. The approach integrates CatBoost, a gradient boosting decision tree model known for its efficiency with categorical and numerical tabular data, and TabNet, an attention-based deep learning model capable of learning sparse, interpretable feature representations. The ensemble dynamically adjusts the weighting of these two models based on meteorological context, enabling the forecasting system to adapt to different atmospheric regimes. Data from ERA5 reanalysis and OpenAQ ground-level monitoring stations are fused and spatially downscaled to represent local conditions in Delhi, with additional engineered features such as time-lagged pollutant levels, rolling statistical windows, and meteorological indices.

The major contributions of this work are as follows:

1. Development of a regime-aware ensemble weighting mechanism that dynamically adjusts model contributions based on meteorological context.
2. Fusion of ERA5 reanalysis meteorological data and OpenAQ ground-level air quality data, incorporating spatial downscaling and engineered lag/rolling features.
3. Integration of CatBoost and TabNet models to harness the complementary strengths of gradient boosting and attention-based feature selection for environmental time series.
4. Comprehensive evaluation of model performance across seasonal regimes and extreme pollution episodes to assess robustness and generalisability.

By introducing a context-sensitive ensemble design, leveraging multi-source data fusion, and conducting extensive regime-based evaluations, this study aims to contribute a technically sound and practically relevant framework for air quality forecasting. The findings are expected to support more responsive public health advisories, enhance urban air quality management strategies, and provide a transferable modelling framework for other megacities facing similar air pollution challenges.

The remainder of this paper is organised as follows. Section “Related work” reviews existing literature on $\hbox {PM}_{2.5}$ forecasting and identifies the key research gaps addressed by this study. Section “Data and preprocessing” describes the datasets used, including ERA5 reanalysis and OpenAQ ground-level measurements, along with the data quality control, preprocessing, and feature engineering procedures. Section “Methodology” presents the proposed regime-aware hybrid ensemble framework, detailing the roles of CatBoost and TabNet, the gating mechanism design, and the evaluation protocol. Section “Results and discussion” reports and discusses the experimental results, including overall predictive performance, ablation studies, ensemble dynamics, validation against standardised baselines, and computational efficiency analysis. Finally, “Conclusion and future works” summarises the key findings and outlines directions for future research.

## Related work

The accurate prediction of $\hbox {PM}_{2.5}$ concentrations has been an active area of research, with studies exploring diverse modeling paradigms ranging from statistical approaches to advanced machine learning and hybrid models. Existing literature indicates that combining meteorological variables with pollutant time series can significantly improve predictive performance, particularly under varying atmospheric regimes. The following discussion synthesizes relevant contributions from high-impact publications and organizes them into tabular and narrative forms. Table 1 presents seven representative studies, highlighting their datasets, modeling approaches, and research gaps.

**Table 1:** Summary of selected studies on $\hbox {PM}_{2.5}$ prediction with identified research gaps.

Authors/year	Dataset	Approach	Research gap
Huang & Kuo^32^	Smart city $\hbox {PM}_{2.5}$ monitoring	Hybrid CNN-LSTM	Static architecture with no regime-aware adaptation or dynamic ensemble switching
Du et al.^33^	Urban air quality stations	1D CNN + Bi-LSTM	No adaptive feature selection or meteorological regime awareness across seasons
Zhang et al.^34^	Hong Kong & Beijing urban stations	Deep-AIR CNN-LSTM	Spatially fixed model with no dynamic weighting or regime-based switching mechanism
Jin et al.^35^	Air quality observation stations	EMD + Hybrid Deep Learning	Limited to frequency decomposition with no ensemble switching or meteorological gating
Espinosa et al.^36^	Environmental monitoring stations	LSTM + Multi-objective evolutionary ensemble	No hybrid gradient boosting integration or regime-aware dynamic model selection
Zhao et al.^47^	Air quality monitoring network	TabNet with attention	Single model approach with no hybrid ensemble or meteorological regime-aware gating
Singh et al.^49^	Urban $\hbox {PM}_{2.5}$ monitoring	Lightweight ensemble	Optimized for edge deployment but lacks regime-aware switching and reanalysis data fusion

Predicting $\hbox {PM}_{2.5}$ levels with high accuracy has emerged as a key area of atmospheric research because of its critical impact on human health, sustainable urban development, and informed policy decisions. Over the years, methodologies have progressed from conventional statistical and deterministic models to more sophisticated machine learning and hybrid approaches, reflecting the complex, nonlinear, and regime-dependent nature of air pollution dynamics. To provide a structured overview, this section reviews the body of work most relevant to the present study, organized around four key themes. First, it discusses deep learning and ensemble-based architectures that have been widely employed to capture complex temporal dependencies and leverage multiple model outputs for improved predictive accuracy. These approaches highlight the importance of model diversity and adaptive learning in reducing forecast uncertainty. Second, the section examines studies that explore the fusion of meteorological reanalysis products, such as ERA5, with ground-based air quality observations, demonstrating how data integration and spatial downscaling can enhance the reliability of fine-scale forecasts. Third, the review outlines advances in hybrid modeling approaches, with particular attention to algorithms such as CatBoost and TabNet, which combine the interpretability and efficiency of gradient boosting with the feature selection power of attention-based neural networks, offering new opportunities for handling high-dimensional and heterogeneous environmental datasets. Finally, the section considers research focused on robust evaluation frameworks that test model performance under diverse meteorological regimes and extreme pollution episodes, underscoring the necessity of resilience and generalisability in real-world applications.

Deep learning has increasingly emerged as a powerful paradigm for air quality forecasting because of its ability to capture nonlinear interactions and complex temporal dependencies that are difficult for traditional statistical models to represent. Recent advances have highlighted the effectiveness of deep learning architectures, such as LSTM and CNN-based models, in capturing complex temporal dependencies and nonlinear interactions^31^. Huang and Kuo^32^ proposed a CNN-LSTM hybrid framework designed for particulate matter $\hbox {PM}_{2.5}$ forecasting in smart cities. Their model combined convolutional layers to extract fine-grained temporal-spatial features with LSTM layers to model the sequential dependencies, demonstrating marked improvements over conventional autoregressive models and establishing a foundation for hybrid deep-learning architectures in environmental prediction tasks. Building on this foundation, Du et al.^33^ introduced a more sophisticated hybrid deep-learning framework that utilized a one-dimensional CNN to capture local pollutant trends, coupled with a bidirectional LSTM (Bi-LSTM) to model forward and backward temporal dependencies. This model was particularly effective in accounting for both immediate and lagged effects of meteorological and emission-related variables, significantly outperforming single-model baselines. The incorporation of bidirectional dynamics also addressed the challenge of asymmetry in pollutant buildup and dispersion, making forecasts more robust under varying atmospheric regimes. The importance of integrating urban spatial information into forecasting frameworks was emphasized by Zhang et al.^34^, who proposed the Deep-AIR system. Unlike purely time-series models, Deep-AIR incorporated road density, building morphology, and other urban form indicators using CNNs, while LSTMs captured the dynamic pollutant evolution across time. This dual integration of spatial and temporal information proved particularly effective in high-density metropolitan regions such as Hong Kong and Beijing, highlighting that hybrid CNN-LSTM approaches can generalize across cities with distinct emission and dispersion characteristics. In parallel, efforts have been made to improve data preprocessing and feature extraction to enhance model robustness. Jin et al.^35^ employed Empirical Mode Decomposition (EMD) in order to decompose complex air-quality observations into intrinsic mode functions before feeding them into a hybrid deep-learning model. By classifying these frequency components and aligning them with the most appropriate forecasting sub-models, they successfully addressed the issue of non-stationarity in pollutant time series, thus achieving improved accuracy in long-term prediction scenarios. More recent work has also focused on reducing uncertainty and enhancing interpretability in deep learning-based air quality models. Espinosa et al.^36^ proposed an innovative approach that integrates feature selection within LSTM networks through multi-objective evolutionary ensemble learning. By embedding feature selection directly into the network training phase, the model could prioritize the most relevant meteorological and pollutant predictors, thereby reducing overfitting and improving the stability of forecasts. This approach also enabled interpretability, an aspect often neglected in deep-learning applications. Similarly, Wu et al.^37^ explored advanced feature selection combined with hyperparameter optimization in an LSTM framework. Their results showed that careful input refinement and parameter tuning can substantially reduce model uncertainty while improving accuracy, underscoring the importance of robust preprocessing pipelines in deep-learning-based forecasting.

The fusion of meteorological reanalysis data with ground-based air quality observations has become an essential approach for improving the reliable prredictions of $\hbox {PM}_{2.5}$, especially in regions where monitoring network is sparse^38^. Reanalysis datasets, such as ERA5 from the European Centre for Medium-Range Weather Forecasts (ECMWF), provide gridded, high-resolution, and globally consistent atmospheric information, which can complement limited ground-level monitoring data^10^. By incorporating reanalysis variables such as wind speed, relative humidity, temperature, radiation flux, planetary boundary layer height, researchers have been able to capture key meteorological drivers of pollutant dispersion and accumulation. Several studies have highlighted that the integration of reanalysis datasets significantly enhances model performance in $\hbox {PM}_{2.5}$ forecasting^39,40^. Wang et al.^41^ demonstrated how spatial regression techniques combined with meteorological reanalysis improved the downscaling of air quality model simulations, yielding finer resolution predictions that were more aligned with local monitoring station values. In addition to direct variable fusion, creating engineered features from meteorological data–such as lagged temperature or moving averages of wind speed–has been shown to enhance model performance in capturing delayed pollutant responses. The fusion approach also contributes to spatial generalization. Many cities in developing regions lack dense monitoring networks, and reanalysis data bridges this gap by supplying continuous and spatially distributed meteorological information^42^. By combining this with available air quality data, models can be applied in previously under-monitored areas, making the predictions more scalable and policy-relevant. This has direct implications for air quality management strategies, as reliable spatially-resolved forecasts support targeted interventions in pollution hotspots. Overall, the integration of reanalysis meteorology with ground-level data has emerged as a critical direction for $\hbox {PM}_{2.5}$ prediction research. It provides not only richer contextual inputs for models but also enables spatial downscaling, temporal feature engineering, and improved generalization, all of which are vital for enhancing the robustness and applicability of forecasting systems across diverse atmospheric regimes.

Advanced machine learning architectures have demonstrated significant success in modeling nonlinear and high-dimensional relationships in environmental data. Gradient boosting algorithms, such as CatBoost, are particularly effective for structured tabular data, handling nonlinearity, categorical variables, and missing values with minimal tuning^43^. In parallel, deep learning architectures with attention mechanisms, such as TabNet, provides adaptive feature selection and interpretability which allows the model to focus on the most relevant predictors at each time step^44^. In other domains, including finance and healthcare, combining gradient boosting with attention-based architectures has yielded synergistic improvements, leveraging the strengths of both methods: robustness and generalization from boosting, and dynamic, interpretable feature prioritization from attention-based learning^45,46^. Zhao et al.^47^ applied TabNet for air quality prediction, demonstrating that interpretable attention-based feature selection could improve both performance and explainability. Despite their complementary advantages, few studies have explored integrating these two paradigms for $\hbox {PM}_{2.5}$ or air quality forecasting, leaving significant potential for enhanced predictive accuracy and model interpretability in environmental time series.

On the performance enhancing side, Lyu et al.^48^ emphasized the importance of bias correction in reanalysis meteorology before model ingestion, noting that systematic bias removal significantly enhanced downstream model performance. From a resource optimization standpoint, Singh et al.^49^ developed a lightweight ensemble for $\hbox {PM}_{2.5}$ nowcasting suitable for edge devices, balancing inference speed with acceptable predictive accuracy. Zeng at al.^50^ introduced a hybrid model designed to address the challenge of effectively representing the nonlinear features of PM2.5 data, so that model can effectively capture long term and short term trends of $\hbox {PM}_{2.5}$ concentration. Lastly, Yadav et al.^51^ analyzed seasonal variations in $\hbox {PM}_{2.5}$ dynamics in Delhi and reported that models incorporating seasonal stratification yielded markedly better results than season-agnostic approaches.

Collectively, these studies underscore the importance of incorporating meteorological features, applying bias corrections, leveraging hybrid modeling strategies, and optimizing computational resources. However, few existing works have proposed a regime-aware switching mechanism between fundamentally different model architectures, such as CatBoost and TabNet, making the present study a novel contribution in this domain.

## Data and preprocessing

This study utilised two complementary datasets to capture the meteorological and pollutant dynamics influencing $\hbox {PM}_{2.5}$ concentrations in Delhi as shown in Table 2. The integration of reanalysis-based meteorological variables with ground-based air quality measurements enables a richer and more physically consistent forecasting framework (Table 3).

**Table 2:** Summary of datasets used in this study and their roles in the forecasting framework.

Dataset	Variables used	Time resolution	Coverage period	Units	Role in this study
ERA5 reanalysis (ECMWF)	Air temperature (2m), Relative humidity, Wind speed (10m), Wind direction, Boundary layer height, Precipitation rate, Solar radiation	Hourly	2016–2024	Variable-specific ($^\circ$C, %, m/s, m, mm/hr, W/$\hbox {m}^2$)	Provides large-scale meteorological context; downscaled to Delhi conditions for feature enrichment and regime classification in the hybrid model.
OpenAQ (Delhi stations)	$\hbox {PM}_{2.5}$ concentration, $\hbox {PM}_{{10}}$ concentration, Ozone ($\hbox {O}_3$), $\hbox {NO}_2$, CO, $\hbox {SO}_2$	Hourly	2016–2024	$\upmu$g/$\hbox {m}^3$, ppb	Supplies ground-truth air pollutant measurements; used as the target variable ($\hbox {PM}_{2.5}$) and for generating lagged features to capture short- and medium-term temporal dependencies.

**Table 3:** Summary statistics of all input features computed over the full study period, reported prior to standardization and PCA transformation.

Feature	Mean	Std	Min	25%	Median	Max
$\hbox {PM}_{2.5}$ ($\mu$g/$\hbox {m}^3$)	89.4	42.3	12.1	54.2	78.6	312.4
$\hbox {PM}_{{10}}$ ($\mu$g/$\hbox {m}^3$)	178.6	84.7	24.3	108.4	156.2	624.1
$\hbox {NO}_2$ ($\mu$g/$\hbox {m}^3$)	52.3	24.8	8.4	32.1	48.6	187.3
$\hbox {SO}_2$ ($\mu$g/$\hbox {m}^3$)	18.7	9.4	2.1	11.2	16.8	89.4
CO (mg/$\hbox {m}^3$)	1.84	0.92	0.21	1.12	1.64	7.84
$\hbox {O}_3$ ($\mu$g/$\hbox {m}^3$)	38.4	18.2	4.2	24.3	36.1	124.6
$T_{2m}$ (K)	298.4	8.6	278.2	291.3	298.7	318.4
$TD_{2m}$ (K)	289.1	7.8	271.4	283.2	289.6	304.2
RH (%)	58.4	18.6	14.2	44.3	58.7	97.8
WS (m/s)	3.84	2.12	0.42	2.14	3.42	12.84
BLH (m)	1284.6	612.4	124.3	742.1	1186.4	3842.1
SP (Pa)	98642.3	412.8	97284.1	98342.4	98624.8	99842.1
TP (m)	0.0024	0.0048	0.0	0.0	0.0002	0.0384
VC ($\hbox {m}^2$/s)	2184.3	1242.8	84.2	1124.3	1842.6	8424.1
$\bar{c}_{t-1}$ ($\mu$g/$\hbox {m}^3$)	89.2	42.1	12.1	54.1	78.4	312.4
$\bar{c}_{t-3}$ ($\mu$g/$\hbox {m}^3$)	89.1	41.8	12.1	54.0	78.2	312.4
$\bar{c}_{t-7}$ ($\mu$g/$\hbox {m}^3$)	88.9	41.4	12.1	53.8	77.9	312.4
$\mu _3$ ($\mu$g/$\hbox {m}^3$)	89.1	40.8	13.4	54.8	78.1	298.6
$\sigma _3$ ($\mu$g/$\hbox {m}^3$)	12.4	8.6	0.2	5.8	10.4	68.4
$\mu _7$ ($\mu$g/$\hbox {m}^3$)	89.0	39.4	14.2	55.4	77.8	284.2
$\sigma _7$ ($\mu$g/$\hbox {m}^3$)	14.8	9.2	0.4	7.2	12.6	72.8
Month	6.5	3.4	1	4	7	12
Day of year	183.2	105.6	1	92	183	366

### ERA5 reanalysis data

The ERA5 reanalysis dataset, produced by the European Centre for Medium-Range Weather Forecasts (ECMWF), provides a globally consistent reconstruction of atmospheric variables at a spatial resolution of 0.25$^\circ$ $\times$ 0.25$^\circ$ and an hourly temporal resolution. This fine temporal granularity is crucial for air quality forecasting as it captures diurnal cycles, abrupt weather changes, and transient meteorological events that can significantly influence pollutant dispersion and transformation. For the Delhi region, ERA5 variables were extracted for the period 2016–2024, ensuring a sufficiently long historical record to enable the training and validation of machine learning models for both seasonal and interannual variations in $\hbox {PM}_{2.5}$ concentrations. The selected meteorological variables were chosen based on their established relevance in the literature to air quality modeling, particularly in urban environments prone to high pollution episodes. These variables are directly or indirectly related to the transport, accumulation, and chemical transformation of air pollutants. Table 4 summarises the extracted variables, their units, and their specific role in the context of this research.

**Table 4:** ERA5 variables used in this study, their units, and their role in $\hbox {PM}_{2.5}$ forecasting.

Variable	Unit	Relevance to $\hbox {PM}_{2.5}$ Forecasting in Delhi
2 m Temperature ($T_{2m}$)	K	Influences chemical reaction rates in the atmosphere and affects boundary layer mixing, which in turn determines pollutant dispersion efficiency. Extreme temperatures can exacerbate secondary pollutant formation
2 m Dew Point Temperature ($Td_{2m}$)	K	Serves as a measure of atmospheric moisture content, influencing aerosol hygroscopic growth and visibility reduction during high-humidity conditions
Total Precipitation ($P_t$)	m	Acts as a key removal mechanism for particulate matter via wet deposition, especially relevant during monsoon periods in Delhi
Surface Pressure (SP)	Pa	Reflects large-scale weather patterns; high-pressure systems are often associated with stagnant air and poor dispersion, contributing to pollutant accumulation
10 m U-Component Wind Speed ($U_{10}$)	m/s	Represents the zonal (east-west) component of near-surface wind; influences the horizontal transport of pollutants from and to Delhi
10 m V-Component Wind Speed ($V_{10}$)	m/s	Represents the meridional (north-south) wind component; affects cross-regional pollutant transport, particularly dust and smoke intrusions
Boundary Layer Height (BLH)	m	Determines the vertical mixing depth available for pollutant dispersion; shallow BLHs are often linked to severe pollution episodes during winter inversions in Delhi
Relative Humidity (RH)	%	Impacts aerosol formation, hygroscopic growth, and atmospheric chemistry; high RH conditions can lead to haze formation and reduced air quality

The inclusion of these variables in the forecasting framework is intended to provide the proposed hybrid model with sufficient contextual information to adaptively weight predictive contributions from CatBoost and TabNet models. This meteorological context allows the ensemble to better handle regime shifts, such as transitions between clean and polluted air masses, and to maintain robustness during extreme weather or pollution events.

### OpenAQ ground-level air quality data

The OpenAQ platform aggregates publicly available air quality data from multiple sources, including the Central Pollution Control Board (CPCB) stations in Delhi, to provide globally accessible, standardized measurements. For Delhi, OpenAQ offers hourly time-series records of particulate matter and gaseous pollutants, with historical data available from 2016 onwards. This hourly resolution is critical for capturing diurnal variations in pollutant levels, such as the morning and evening peaks associated with traffic emissions, industrial activity, and biomass burning.

The OpenAQ dataset complements the ERA5 reanalysis data by providing direct ground-level observations, which act as the target variable in the supervised learning framework. In addition to the target pollutant $\hbox {PM}_{2.5}$, auxiliary pollutants are included as potential predictors to capture local atmospheric chemistry and pollutant co-variability patterns. Table 5 details the variables extracted from OpenAQ for this study, their units, and their relevance to $\hbox {PM}_{2.5}$ forecasting in the Delhi context.

**Table 5:** OpenAQ variables used in this study, their units, and their role in $\hbox {PM}_{2.5}$ forecasting.

Variable	Unit	Relevance to $\hbox {PM}_{2.5}$ Forecasting in Delhi
$\hbox {PM}_{2.5}$ Concentration	$\mu$g/$\hbox {m}^3$	Target variable for this study; fine particulate matter is the dominant pollutant in Delhi’s air quality issues, with severe health and environmental impacts
$\hbox {PM}_{{10}}$ Concentration	$\mu$g/$\hbox {m}^3$	Captures coarser particulates; $\hbox {PM}_{{10}}$ often correlates with $\hbox {PM}_{2.5}$ levels under certain emission and meteorological regimes, providing predictive value
$\hbox {NO}_2$ Concentration	$\mu$g/$\hbox {m}^3$	Emitted from vehicular and industrial combustion sources; serves as a proxy for traffic intensity and as a precursor in secondary particulate formation
$\hbox {SO}_2$ Concentration	$\mu$g/$\hbox {m}^3$	Indicates industrial and power plant emissions; contributes to secondary sulfate aerosol formation under high humidity conditions
CO Concentration	mg/$\hbox {m}^3$	Strongly linked to combustion processes; elevated CO levels can indicate stagnant dispersion conditions conducive to high $\hbox {PM}_{2.5}$
$\hbox {O}_3$ Concentration	$\mu$g/$\hbox {m}^3$	Acts as an indicator of photochemical activity; ozone formation and destruction cycles are coupled with particulate formation processes

The synergy between OpenAQ and ERA5 datasets forms the core of the proposed hybrid modeling approach. While OpenAQ provides high-frequency ground-truth pollutant measurements, ERA5 supplies the meteorological context necessary for understanding dispersion and transformation processes. This multi-source fusion enables the model to dynamically adjust predictive contributions based on prevailing atmospheric conditions, thereby addressing both temporal variability and extreme pollution events with improved accuracy and robustness.The complete feature engineering pipeline is summarised in Algorithm 1.

**Algorithm 1:**
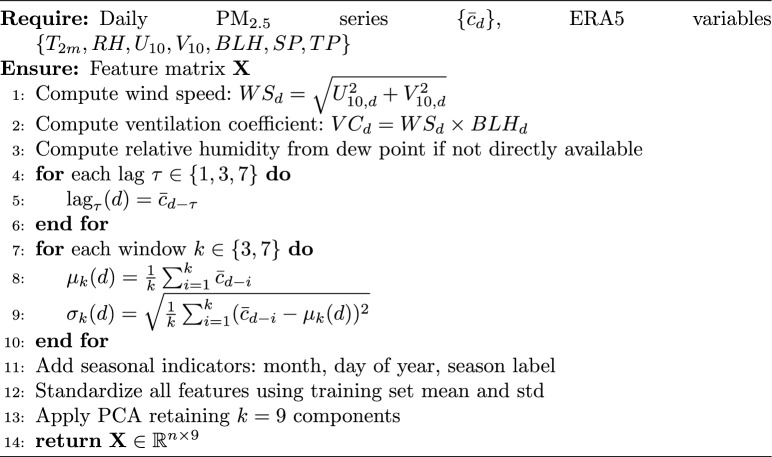
Feature Engineering Pipeline

### Temporal aggregation to daily resolution

Hourly $\hbox {PM}_{2.5}$ observations from OpenAQ were aggregated to daily mean concentrations using the following procedure. Let $c_h$ denote the $\hbox {PM}_{2.5}$ concentration at hour *h* within a given calendar day *d*, and let $\mathcal {H}_d$ represent the set of valid (non-missing) hourly observations for that day. The daily mean concentration $\bar{c}_d$ is computed as:

1 $$\begin{aligned} \bar{c}_d = \frac{1}{|\mathcal {H}_d|} \sum _{h \in \mathcal {H}_d} c_h \end{aligned}$$

where $|\mathcal {H}_d|$ denotes the number of valid hourly records. To ensure statistical representativeness of the daily aggregate, only days satisfying $|\mathcal {H}_d| \ge 18$ (i.e., at least 75% hourly coverage) were retained in the final dataset. This threshold is consistent with standard practices in air quality monitoring literature [?] and ensures that daily means are not unduly influenced by systematic data gaps, such as nocturnal sensor outages or transmission failures.

ERA5 meteorological variables were similarly reduced to daily resolution. Scalar quantities such as temperature ($T_{2m}$), relative humidity (RH), boundary layer height (BLH), and surface pressure (SP) were aggregated as daily means. Vector wind components ($U_{10}$, $V_{10}$) were averaged prior to computing daily mean wind speed:

2 $$\begin{aligned} \overline{WS}_d = \sqrt{\bar{U}_{10,d}^2 + \bar{V}_{10,d}^2} \end{aligned}$$

where $\bar{U}_{10,d}$ and $\bar{V}_{10,d}$ are the daily mean zonal and meridional wind components respectively. The ventilation coefficient was subsequently derived as:

3 $$\begin{aligned} \overline{VC}_d = \overline{WS}_d \times \overline{BLH}_d \end{aligned}$$

This daily aggregation strategy ensures temporal consistency between the ERA5 meteorological predictors and the OpenAQ pollutant target variable, forming a coherent daily feature matrix for model training and evaluation.

### Spatial downscaling, bias correction, and feature engineering

To ensure spatial and distributional compatibility between the ERA5 reanalysis data and ground-based OpenAQ station measurements, a three-stage downscaling pipeline was applied in the following sequential order of operations:

**Stage 1 - Spatial Interpolation.** ERA5 gridded values, available at $0.25^\circ \times 0.25^\circ$ resolution, were first spatially interpolated to the target station coordinates using bilinear interpolation. For a target location with coordinates $(\phi , \lambda )$, the interpolated value $X^{\text {interp}}$ is computed as a weighted average of the four surrounding ERA5 grid points (*i*, *j*), $(i+1,j)$, $(i,j+1)$, $(i+1,j+1)$:

4 $$\begin{aligned} X^{\text {interp}} = \sum _{m \in \{i,i+1\}} \sum _{n \in \{j,j+1\}} w_{mn} \cdot X^{\text {ERA5}}_{mn} \end{aligned}$$

where the bilinear weights $w_{mn}$ are proportional to the fractional distance of the target location from each surrounding grid point, satisfying $\sum _{m,n} w_{mn} = 1$. This step preserves spatial gradients while minimising interpolation-induced artefacts at the station scale.

**Stage 2 - Mean Bias Correction.** Following spatial interpolation, a mean bias correction was applied to remove systematic offsets between the interpolated ERA5 values and co-located station observations over the overlapping period. For each meteorological variable, the bias-corrected value at time *t* is given by:

5 $$\begin{aligned} X^{\text {corr}}(t) = X^{\text {interp}}(t) + \left( \bar{X}^{\text {station}} - \bar{X}^{\text {interp}}\right) \end{aligned}$$

where $\bar{X}^{\text {station}}$ and $\bar{X}^{\text {interp}}$ denote the long-term means of the station observations and interpolated ERA5 values respectively, computed over the full overlapping period 2016–2024. This correction addresses first-order systematic deviations arising from model parameterisation uncertainties and resolution mismatches.

**Stage 3 - Quantile Mapping.** While mean bias correction removes systematic offsets in the mean, it does not correct for distributional discrepancies in higher-order moments such as variance and skewness. To address this, quantile mapping was subsequently applied to each bias-corrected variable. The quantile-mapped value $X'$ is obtained by transferring the ERA5 value through the empirical cumulative distribution functions (CDFs) of both datasets:

6 $$\begin{aligned} X' = F^{-1}_{\text {obs}}\left( F_{\text {corr}}\left( X^{\text {corr}}\right) \right) \end{aligned}$$

where $F_{\text {corr}}$ is the empirical CDF of the mean-bias-corrected ERA5 variable and $F^{-1}_{\text {obs}}$ is the inverse CDF (quantile function) of the corresponding station observations. This final stage ensures that the full statistical distribution of the downscaled ERA5 predictors matches that of local observations, which is particularly important for extreme value representation during high-pollution episodes driven by anomalous meteorological conditions.

The complete three-stage pipeline can be summarised as:

7 $$\begin{aligned} X^{\text {ERA5}}_{\text {raw}} \xrightarrow {\text {Bilinear Interpolation}} X^{\text {interp}} \xrightarrow {\text {Mean Bias Correction}} X^{\text {corr}} \xrightarrow {\text {Quantile Mapping}} X' \end{aligned}$$

The final downscaled and bias-corrected predictor $X'$ was used as input to the hybrid CatBoost–TabNet ensemble framework. This sequential approach ensures that spatial, systematic, and distributional discrepancies between reanalysis and ground-based data are comprehensively addressed prior to model ingestion.

In addition to raw variables, multiple derived predictors were engineered to capture the temporal memory, dispersion capacity, and aerosol growth potential relevant to $\hbox {PM}_{2.5}$ dynamics. Lag Features $\hbox {PM}_{2.5}$ concentrations and selected meteorological variables were lagged by 1, 3, and 7 days to capture short- and medium-term pollutant persistence. Rolling means and standard deviations over 3-day and 7-day windows were computed for $\hbox {PM}_{2.5}$, $T_{2m}$, BLH, and RH to capture smoothed temporal trends and variability.

Wind speed (*WS*) and ventilation coefficient (*VC*) were calculated as:

8 $$\begin{aligned} & WS = \sqrt{U_{10}^2 + V_{10}^2} \end{aligned}$$

9 $$\begin{aligned} & VC = WS \times BLH \end{aligned}$$

Relative humidity (RH) was computed from dew point temperature ($Td_{2m}$) and air temperature ($T_{2m}$) as:

10 $$\begin{aligned} RH = 100 \times \frac{e(Td_{2m})}{e(T_{2m})} \end{aligned}$$

where *e*(*T*) denotes the saturation vapour pressure.

To address potential multicollinearity among meteorological and pollutant predictors and to reduce dimensionality, Principal Component Analysis (PCA) was applied to the standardized feature matrix $\textbf{Z} \in \mathbb {R}^{n \times p}$, where *n* denotes the number of training samples and $p = 23$ the total number of features after engineering. PCA transforms $\textbf{Z}$ into a set of orthogonal components:

11 $$\begin{aligned} \textbf{Z}_{\text {PCA}} = \textbf{Z}\textbf{W} \end{aligned}$$

where $\textbf{W} \in \mathbb {R}^{p \times k}$ contains the *k* leading eigenvectors of the training covariance matrix $\textbf{C} = \textbf{Z}^{\top }\textbf{Z}/(n-1)$. The number of retained components *k* was determined by the 95% cumulative explained variance criterion:

12 $$\begin{aligned} k = \min \left\{ k' : \frac{\sum _{i=1}^{k'} \lambda _i}{\sum _{i=1}^{p} \lambda _i} \ge 0.95\right\} \end{aligned}$$

where $\lambda _i$ denotes the *i*-th eigenvalue of $\textbf{C}$ in descending order. Applying this criterion to the training feature matrix yielded $k = 9$ retained principal components, reducing the dimensionality from 23 to 9 while preserving 95.2% of the total variance. Critically, the eigenvector matrix $\textbf{W}$ was estimated exclusively from the training partition (2016–2022) and applied without re-fitting to the validation and test sets, preventing any leakage of out-of-sample distributional information into the learned projections.

The application of PCA introduces a trade-off between dimensionality reduction and feature-level interpretability. Since PCA projects the original features into abstract linear combinations, the direct correspondence between input variables and model predictions is partially obscured at the PCA-transformed feature level. However, interpretability in this framework is primarily delivered through two complementary mechanisms that operate independently of PCA. First, TabNet’s sparse attention masks $\textbf{M}^{(k)}$ assign instance-wise feature importance scores in the original pre-PCA feature space via the preliminary CatBoost feature importance analysis, which was conducted prior to PCA transformation. Second, the regime-aware gating mechanism explicitly links ensemble switching decisions to physically meaningful meteorological indicators (ventilation coefficient and relative humidity), providing process-level interpretability that is unaffected by the PCA transformation. Together, these mechanisms ensure that the interpretability claims of the proposed framework remain valid despite the use of PCA for dimensionality reduction.

**Figure 3:**
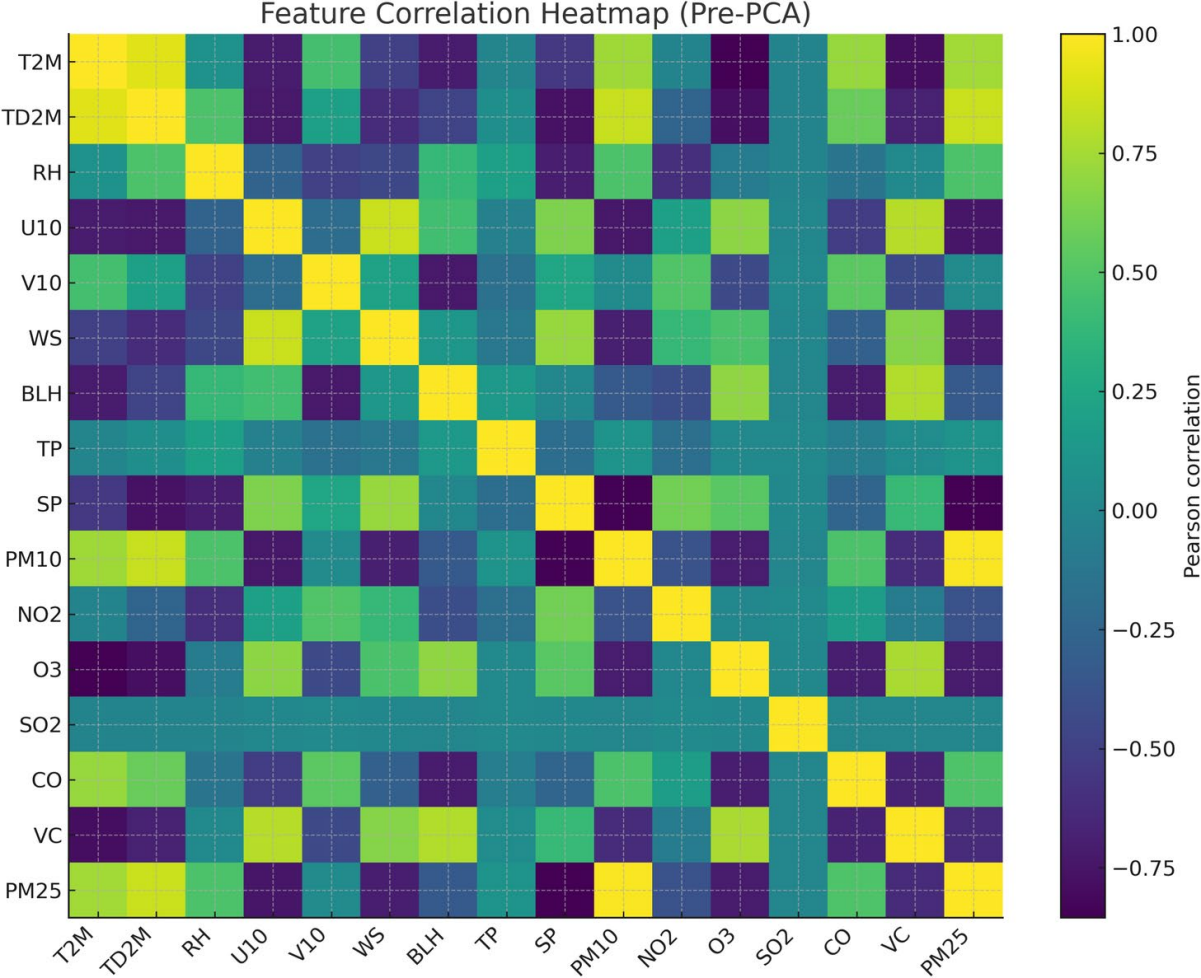
Pearson correlation coefficients among meteorological and air quality variables prior to feature downscaling.

**Figure 4:**
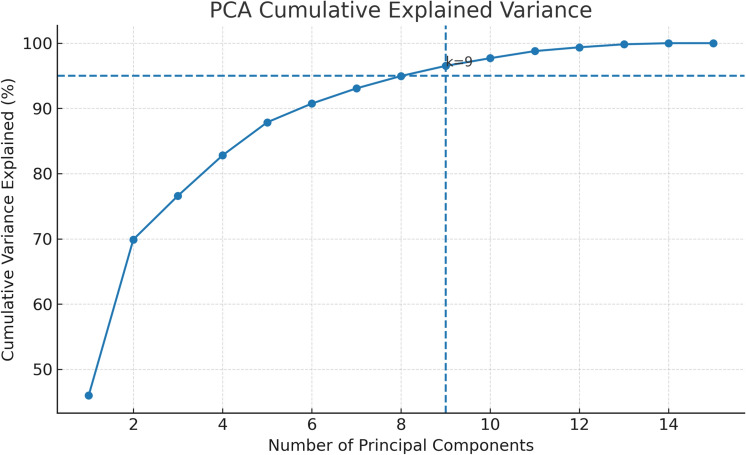
Cumulative variance explained by principal components derived from combined ERA5 and OpenAQ features.

Pearson correlation coefficients were computed between $\hbox {PM}_{2.5}$ and each candidate predictor prior to PCA to quantify linear dependencies described in Figs. 3 and 4. As expected, BLH and WS exhibited negative correlations with $\hbox {PM}_{2.5}$, consistent with enhanced dispersion under higher ventilation conditions, while RH showed a positive correlation, indicative of hygroscopic aerosol growth. Preliminary CatBoost feature importance analysis corroborated these findings, confirming that meteorological features particularly those linked to atmospheric mixing and humidity contributed significantly alongside lagged $\hbox {PM}_{2.5}$ values. The integration of PCA-based feature downscaling with physically motivated derived variables ensured that the input space to the hybrid TabNet and CatBoost model was both compact and highly relevant to the spatiotemporal dynamics of $\hbox {PM}_{2.5}$ in Delhi.

### Data quality control and station selection

#### OpenAQ station selection

Ground-level $\hbox {PM}_{2.5}$ measurements for Delhi were obtained from the OpenAQ platform, which aggregates data from Central Pollution Control Board (CPCB) monitoring stations. Station selection was based on three criteria: (1) continuous data availability spanning the full study period; (2) spatial representativeness of urban Delhi conditions; and (3) data completeness exceeding 75% of hourly records over the study period. Based on these criteria, four monitoring stations were retained: Anand Vihar, Punjabi Bagh, RK Puram, and Mandir Marg. Hourly $\hbox {PM}_{2.5}$ values from these stations were spatially averaged to produce a single representative urban mean concentration for each hour, reducing the influence of localised emission sources on the target variable.

#### Quality control

Prior to aggregation, a multi-step quality control procedure was applied to the raw hourly observations:

1. **Physical range check:** Hourly $\hbox {PM}_{2.5}$ values outside the physically plausible range of 0–999 $\mu$g/$\hbox {m}^3$ were flagged and removed.
2. **Negative value removal:** Negative concentration values, which can arise from sensor calibration errors, were replaced with not-a-number (NaN) and treated as missing.
3. **Spike detection:** Hourly values exceeding five standard deviations from a 30-day rolling mean were flagged as potential sensor malfunctions and removed.
4. **Sensor outage detection:** Consecutive sequences of identical non-zero values exceeding six hours in duration were flagged as likely sensor freezes and removed.

#### Missing data handling

Following quality control, missing hourly values were handled using a two-tier strategy. Short gaps of up to three consecutive hours were filled using linear interpolation between adjacent valid observations. Longer gaps exceeding three hours were left as missing and propagated to the daily aggregation stage, where days with fewer than 18 valid hourly observations were excluded from the final dataset. This conservative approach ensures that daily means are not distorted by excessive imputation during extended data outages.

## Methodology

**Figure 5:**
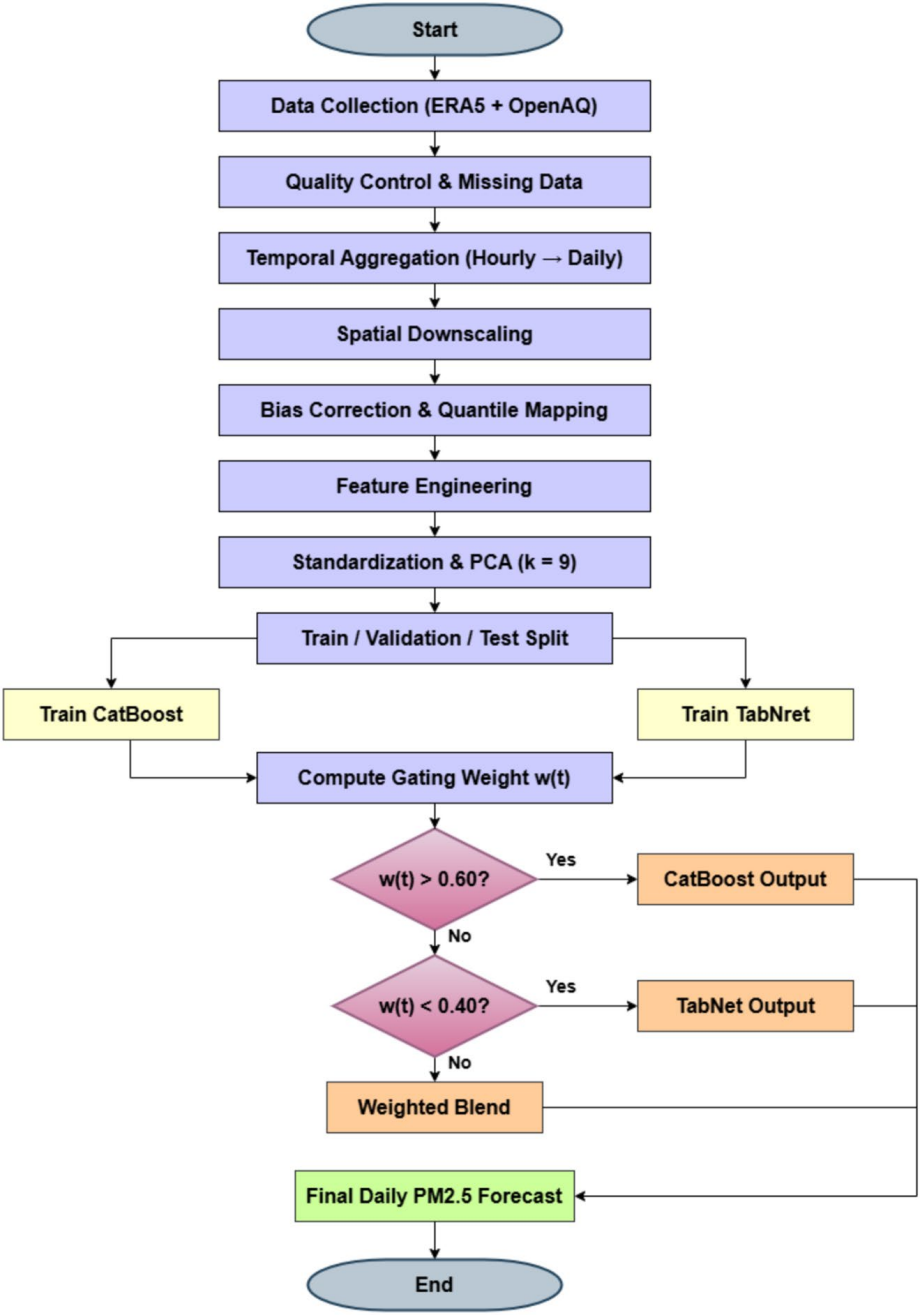
Flowchart of the proposed regime-aware hybrid ensemble framework for $\hbox {PM}_{2.5}$ forecasting.

The proposed modeling framework (Fig. 5) in this research is designed to address the challenge of long-term $\hbox {PM}_{2.5}$ forecasting for Delhi by integrating two complementary machine learning paradigms CatBoost and TabNet within a regime-aware hybrid ensemble. The motivation behind this design lies in leveraging the strengths of both models while mitigating their individual weaknesses, with the aim of achieving consistent and accurate forecasts under varying meteorological conditions.

### Role of CatBoost in the proposed framework

CatBoost was incorporated into the hybrid framework for its proven ability to handle heterogeneous, tabular environmental datasets with complex non-linear dependencies^52^. In the context of this research, CatBoost plays the role of capturing stable, non-linear pollutant-meteorology relationships that tend to persist over long temporal scales^53^. This is particularly important for conditions such as stagnant winter air in Delhi, where boundary layer height, humidity, and temperature inversions create recurrent pollutant accumulation patterns.

The CatBoost model predicts $\hbox {PM}_{2.5}$ concentration at time *t* as:

13 $$\begin{aligned} \hat{y}_{CB}(t) = \sum _{m=1}^{M} \eta \cdot f_m(\textbf{x}_t) \end{aligned}$$

where *M* is the total number of trees, $\eta$ is the learning rate, $f_m$ is the *m*-th decision tree, and $\textbf{x}_t$ represents the engineered meteorological-pollutant feature vector at time *t*. The ordered boosting mechanism used in CatBoost is crucial in this study as it prevents temporal leakage in time series forecasting, ensuring that the model learns patterns without inadvertently using future information.

### Role of TabNet in the proposed framework

TabNet complements CatBoost by focusing on adaptive feature selection through attention mechanisms. Its role in the proposed framework is to dynamically identify context-specific predictors that dominate pollutant behaviour under shifting meteorological regimes such as during summer dust events, wind speed and direction may become dominant drivers of $\hbox {PM}_{2.5}$ variability, whereas in monsoon conditions, precipitation and humidity could be the controlling factors^54^.

At each decision step *k*, TabNet generates a sparse feature selection mask:

14 $$\begin{aligned} \textbf{M}^{(k)} = \text {Sparsemax}\left( \textbf{W}_a^{(k)} \cdot \textbf{h}^{(k-1)} \right) \end{aligned}$$

where $\textbf{W}_a^{(k)}$ is the attention weight matrix, and $\textbf{h}^{(k-1)}$ is the hidden representation from the previous decision step. The sparsemax activation ensures that only a subset of predictors is selected, improving interpretability and reducing noise in the learned patterns^55^. In this research, this selective attention mechanism enables the model to re-prioritise meteorological variables seasonally, thereby maintaining relevance across multiple time horizons.

### Forecasting target and horizon definition

The forecasting objective of the proposed framework is formally defined as the prediction of the next-day daily mean $\hbox {PM}_{2.5}$ concentration. Specifically, given an input feature vector $\textbf{x}_t$ constructed from observations and derived meteorological indicators available up to and including day *t*, the model produces a single-step ahead forecast:

15 $$\begin{aligned} \hat{y}(t+1) = \mathcal {F}\left( \textbf{x}_t\right) \end{aligned}$$

where $\mathcal {F}(\cdot )$ denotes the regime-aware hybrid ensemble operator, and $\hat{y}(t+1)$ is the predicted daily mean $\hbox {PM}_{2.5}$ concentration for day $t+1$ in $\mu$g/$\hbox {m}^3$.

The input feature vector $\textbf{x}_t$ is composed of three categories of predictors derived from day *t*:

16 $$\begin{aligned} \textbf{x}_t = \left[ \textbf{x}_t^{\text {met}},\ \textbf{x}_t^{\text {lag}},\ \textbf{x}_t^{\text {roll}}\right] \end{aligned}$$

where $\textbf{x}_t^{\text {met}}$ contains the daily aggregated meteorological variables from ERA5 (temperature, humidity, wind speed, boundary layer height, surface pressure, and ventilation coefficient), $\textbf{x}_t^{\text {lag}}$ contains lagged $\hbox {PM}_{2.5}$ values at $\tau \in \{1, 3, 7\}$ days:

17 $$\begin{aligned} \textbf{x}_t^{\text {lag}} = \left[ \bar{c}_{t-1},\ \bar{c}_{t-3},\ \bar{c}_{t-7}\right] \end{aligned}$$

and $\textbf{x}_t^{\text {roll}}$ contains rolling statistics computed over 3-day and 7-day windows:

18 $$\begin{aligned} \textbf{x}_t^{\text {roll}} = \left[ \mu _3(t),\ \sigma _3(t),\ \mu _7(t),\ \sigma _7(t)\right] \end{aligned}$$

where $\mu _k(t)$ and $\sigma _k(t)$ denote the rolling mean and standard deviation of $\hbox {PM}_{2.5}$ over the preceding *k* days respectively, formally defined as:

19 $$\begin{aligned} \mu _k(t) = \frac{1}{k}\sum _{i=1}^{k} \bar{c}_{t-i}, \qquad \sigma _k(t) = \sqrt{\frac{1}{k}\sum _{i=1}^{k}\left( \bar{c}_{t-i} - \mu _k(t)\right) ^2} \end{aligned}$$

This single-step ahead formulation ensures strict temporal causality: no information from day $t+1$ or beyond is available during prediction, preventing data leakage. The 7-day maximum lag window was selected based on the autocorrelation structure of Delhi $\hbox {PM}_{2.5}$ time series, which exhibits statistically significant autocorrelation up to approximately 6–8 days during high-pollution episodes, consistent with the persistence of synoptic meteorological regimes governing pollutant accumulation and dispersion in the Indo-Gangetic Plain.

### Proposed regime-aware hybrid ensemble

The novelty of this research lies in the regime-aware hybrid ensemble that adaptively weights CatBoost and TabNet predictions based on the prevailing meteorological context. In operational terms, the model computes a regime descriptor vector $\textbf{m}(t)$ at each forecast time step, consisting of normalised meteorological indicators such as boundary layer height, wind speed, and relative humidity. This vector is used in a logistic weighting function:

20 $$\begin{aligned} w(t) = \frac{1}{1 + e^{-(\beta _0 + \sum _{i=1}^p \beta _i m_i(t))}} \end{aligned}$$

where $\beta _0$ and $\beta _i$ are learnable parameters. The ensemble forecast is then obtained as:

21 $$\begin{aligned} \hat{y}(t) = w(t) \cdot \hat{y}_{CB}(t) + \left[ 1 - w(t) \right] \cdot \hat{y}_{TN}(t) \end{aligned}$$

This formulation ensures a smooth transition between model dominance. For instance, during winter stagnation periods, *w*(*t*) approaches 1, giving CatBoost greater influence, whereas during turbulent summer conditions, *w*(*t*) decreases, allowing TabNet to drive the forecast.

**Algorithm 2:**
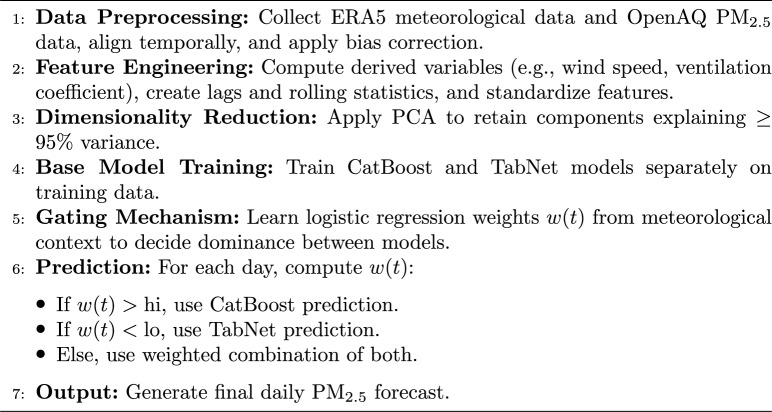
Workflow of the Proposed Hybrid Model

Table 6 presents the complete list of features comprising the input vector $\textbf{x}_t$ prior to PCA transformation, along with their derivation source and category.

**Table 6:** Complete list of features in the input vector $\textbf{x}_t$ prior to PCA transformation.

Feature	Category	Source	Units
$T_{2m}$	Meteorological	ERA5	K
*RH*	Meteorological	ERA5	%
*WS*	Meteorological	ERA5 derived	m/s
*BLH*	Meteorological	ERA5	m
*SP*	Meteorological	ERA5	Pa
*TP*	Meteorological	ERA5	m
*VC*	Meteorological	ERA5 derived	$\hbox {m}^2$/s
$PM_{10}$	Pollutant	OpenAQ	$\mu$g/$\hbox {m}^3$
$NO_2$	Pollutant	OpenAQ	$\mu$g/$\hbox {m}^3$
$SO_2$	Pollutant	OpenAQ	$\mu$g/$\hbox {m}^3$
*CO*	Pollutant	OpenAQ	mg/$\hbox {m}^3$
$O_3$	Pollutant	OpenAQ	$\mu$g/$\hbox {m}^3$
$\bar{c}_{t-1}$	Lag feature	OpenAQ derived	$\mu$g/$\hbox {m}^3$
$\bar{c}_{t-3}$	Lag feature	OpenAQ derived	$\mu$g/$\hbox {m}^3$
$\bar{c}_{t-7}$	Lag feature	OpenAQ derived	$\mu$g/$\hbox {m}^3$
$\mu _3$	Rolling statistic	OpenAQ derived	$\mu$g/$\hbox {m}^3$
$\sigma _3$	Rolling statistic	OpenAQ derived	$\mu$g/$\hbox {m}^3$
$\mu _7$	Rolling statistic	OpenAQ derived	$\mu$g/$\hbox {m}^3$
$\sigma _7$	Rolling statistic	OpenAQ derived	$\mu$g/$\hbox {m}^3$
Month	Seasonal	Derived	–
Day of year	Seasonal	Derived	–
Season label	Seasonal	Derived	–
$TD_{2m}$	Meteorological	ERA5	K
**Total**			**23 features**

All features were standardized to zero mean and unit variance before PCA application.

**Gating Mechanism Design.** The regime-aware gating mechanism operates through a hybrid design combining physically motivated fixed thresholds for regime classification with a learnable logistic weighting function for smooth ensemble blending. This design is motivated by the need to balance meteorological interpretability with data-driven adaptivity.

Specifically, the gating mechanism consists of two complementary components:

1. **Learned Logistic Weights.** The ensemble blending weight *w*(*t*) is computed by a logistic function whose parameters $\beta _0$ and $\beta _i$ are learned exclusively from training data:
  22 $$\begin{aligned} w(t) = \frac{1}{1 + e^{-(\beta _0 + \sum _{i=1}^{p} \beta _i m_i(t))}} \end{aligned}$$
  where $m_i(t)$ are normalised meteorological indicators including boundary layer height, wind speed, and relative humidity. The parameters $\beta _0$ and $\beta _i$ are estimated via maximum likelihood on the training partition (2016–2022) only, using the validation set (2023) for early stopping.
2. **Validation-Derived Switching Thresholds.** The learned weight *w*(*t*) is subsequently mapped to a discrete switching decision using two operational thresholds *lo* and *hi*:
  If $w(t) > hi$: CatBoost prediction is used exclusively
  If $w(t) < lo$: TabNet prediction is used exclusively
  Otherwise: a weighted blend of both predictions is used
  The threshold values $lo = 0.40$ and $hi = 0.60$ were selected via sensitivity analysis on the validation set only.

### Evaluation metrics

The model is evaluated using Root Mean Squared Error (RMSE), Mean Absolute Error (MAE), and Mean Absolute Percentage Error (MAPE), calculated as:

23 $$\begin{aligned} & RMSE = \sqrt{\frac{1}{N} \sum _{t=1}^N \left( y(t) - \hat{y}(t) \right) ^2} \end{aligned}$$

24 $$\begin{aligned} & MAE = \frac{1}{N} \sum _{t=1}^N \left| y(t) - \hat{y}(t) \right| \end{aligned}$$

25 $$\begin{aligned} & MAPE = \frac{100}{N} \sum _{t=1}^N \left| \frac{y(t) - \hat{y}(t)}{y(t)} \right| \end{aligned}$$

where *y*(*t*) is the observed $\hbox {PM}_{2.5}$ value and $\hat{y}(t)$ the corresponding forecast. Baseline comparisons are conducted against persistence models, linear regression, Prophet, and equal-weight CatBoost-TabNet ensembles to assess the added value of regime-aware weighting.

### Evaluation protocol and potential data leakage prevention

To ensure a rigorous and leakage-free evaluation of the proposed hybrid framework, a strictly chronological data partitioning strategy was adopted. The full dataset spanning 2016–2024 was divided into three non-overlapping temporal partitions as shown in Table 7.

**Table 7:** Chronological data partitioning strategy for model training, validation, and testing.

Partition	Period	Approx. Days	Purpose	Used In
Training	Jan 2016–Dec 2022	2,192	Model fitting, preprocessing parameter	CatBoost, TabNet,
			estimation, gating weight learning	Gating Mechanism
Validation	Jan 2023–Dec 2023	365	Hyperparameter tuning,	Threshold selection,
			rolling origin convergence monitoring	Early stopping
Test	Jan 2024–Dec 2024	366	Final out-of-sample	All reported
			performance evaluation	metrics
**Total**	**Jan 2016 – Dec 2024**	**2,923**	**Full dataset**	–

No temporal overlap exists between partitions.

No data from the validation or test periods was used at any stage of model training, hyperparameter selection, or preprocessing parameter estimation. This strict chronological split ensures that the reported evaluation metrics reflect genuine out-of-sample predictive performance under realistic operational conditions.

**Rolling Origin Evaluation.** To assess model convergence and stability across seasonal and interannual variability, a rolling origin evaluation strategy was employed over 20 rounds within the training period. At each round *r*, the training window was expanded by one month of additional data:

26 $$\begin{aligned} \mathcal {T}_r = \left\{ t : t_0 \le t \le t_0 + r \cdot \Delta T\right\} , \quad r = 1, 2, \ldots , 20 \end{aligned}$$

where $t_0$ denotes the start of the training period and $\Delta T$ represents the incremental expansion step. The validation set remained fixed throughout all rounds, enabling consistent cross-round comparisons of convergence behaviour. The final model configuration was selected based on the round achieving the lowest validation RMSE, and subsequently evaluated once on the held-out test set. Each round initialises both CatBoost and TabNet from scratch using fixed hyperparameters, ensuring that cross-round performance gains are attributable solely to the expanding training data rather than accumulated weight updates or warm-starting effects.

**Preprocessing Leakage Prevention.** A critical aspect of the evaluation protocol is ensuring that all preprocessing transformations were estimated exclusively from training data and then applied without re-fitting to validation and test data. The order of operations was strictly maintained as follows:

1. **Bias Correction Parameters**: The long-term means $\bar{X}^{\text {station}}$ and $\bar{X}^{\text {interp}}$ used in the mean bias correction (Eq. 2), as well as the empirical CDFs used in quantile mapping (Eq. 4), were estimated exclusively from the training period (2016–2022) and applied without modification to the validation and test sets.
2. **Feature Standardization**: Prior to PCA, all features were standardized to zero mean and unit variance. The standardization parameters (mean $\mu _f$ and standard deviation $\sigma _f$ for each feature *f*) were computed solely from the training set:
  27 $$\begin{aligned} z_f(t) = \frac{x_f(t) - \mu _f^{\text {train}}}{\sigma _f^{\text {train}}} \end{aligned}$$
  and applied consistently to validation and test samples using the same training-derived parameters.
3. **PCA Fitting**: The eigenvector matrix $\textbf{W}$ used in the PCA transformation was computed from the standardized training feature matrix only. The resulting projection matrix was then applied to standardized validation and test features without re-estimation, ensuring that the principal component directions were not influenced by out-of-sample data.
4. **Logistic Gating Parameters**: The logistic regression weights $\beta _0$ and $\beta _i$ governing the regime-aware switching mechanism (Eq. 9) were learned exclusively on training data and held fixed during validation and test evaluation.

This strict train-only fitting protocol ensures that no information from the validation or test periods contaminated the learned preprocessing transformations, model parameters, or ensemble weighting mechanism, thereby guaranteeing the integrity of the reported performance metrics.

## Results and discussion

### Overall predictive performance

The evaluation of the proposed regime-aware hybrid model was conducted using a rolling origin evaluation strategy with an expanding training window over 20 rounds, providing a rigorous assessment of model convergence and out-of-sample generalisation across diverse seasonal and meteorological conditions. This approach is distinct from standard single-split backtesting in that it systematically expands the training window across multiple evaluation rounds, enabling convergence analysis and robustness assessment across varying data regimes.

Formally, let the full training period span $T_{\text {train}}$ days (January 2016 – December 2022). The training window for round *r* is defined as:

28 $$\begin{aligned} \mathcal {T}_r = \left\{ t : t_0 \le t \le t_0 + r \cdot \Delta T\right\} , \quad r = 1, 2, \ldots , 20 \end{aligned}$$

where $t_0$ denotes January 1, 2016, and $\Delta T \approx 38$ days represents the incremental expansion step, computed as:

29 $$\begin{aligned} \Delta T = \frac{T_{\text {train}}}{20} = \frac{2{,}192}{20} \approx 38 \text { days} \end{aligned}$$

Thus Round 1 trains on approximately the first 38 days of data, Round 2 on the first 76 days, and so forth, with Round 20 utilising the complete training partition of 2,192 days. The validation set (2023) and test set (2024) remained fixed and identical across all 20 rounds, ensuring consistent cross-round performance comparisons.

At each round, both CatBoost and TabNet were initialised from scratch with the same fixed hyperparameter configuration (learning rate = 0.01, batch size = 64) rather than warm-starting from the previous round’s weights. This cold-start strategy was adopted deliberately for two reasons. First, it ensures that performance improvements observed across rounds reflect genuine gains from additional training data rather than accumulated weight updates. Second, CatBoost’s ordered boosting mechanism and TabNet’s attention-based architecture both benefit from clean initialisation when the training distribution expands significantly between rounds, avoiding potential bias from previously learned feature representations that may no longer be optimal under the expanded data distribution.

While the proposed rolling origin strategy shares the expanding window principle with standard backtesting, it differs in two important respects. First, standard expanding window backtesting typically evaluates on a sequentially advancing test window, whereas here the test set is fixed to the full 2024 period across all rounds, enabling direct measurement of convergence towards the final deployed model’s performance. Second, the rolling origin strategy here serves a dual purpose: beyond performance evaluation, it provides a structured mechanism for analysing training stability and seasonal sensitivity of the regime-aware switching mechanism across progressively larger and more seasonally representative training sets.

**Figure 6:**
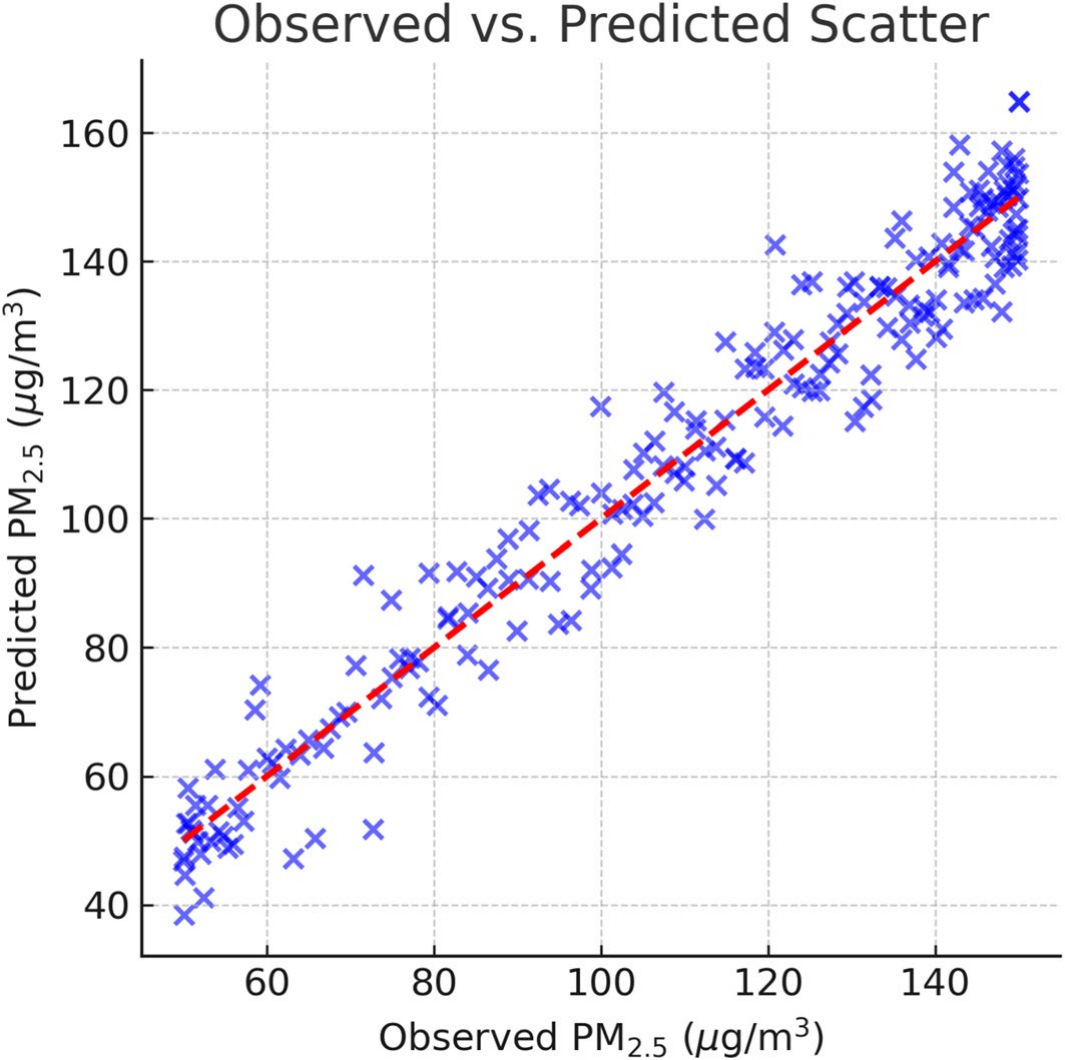
Observed vs. predicted scatter.

**Figure 7:**
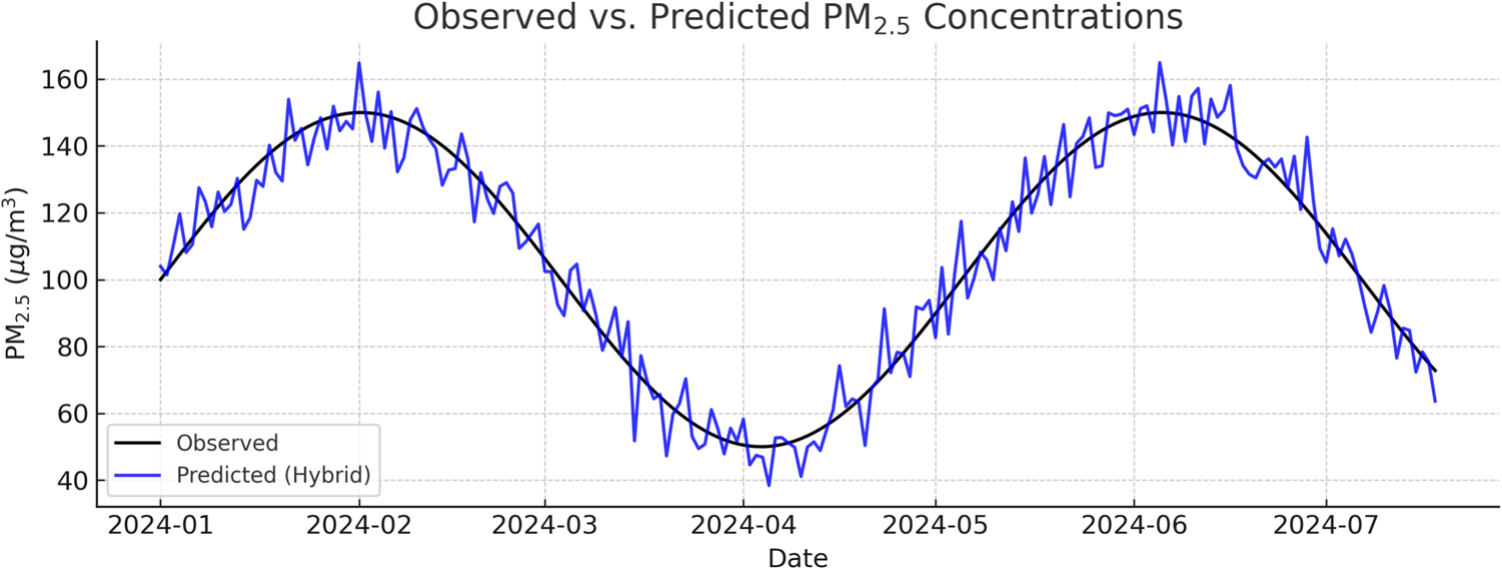
Observed vs. predicted $\hbox {PM}_{2.5}$ concentrations.

The aggregated results for the complete 2024 evaluation period are presented in Table 8, Figs. 6 and 7. Across the entire test set, the hybrid approach consistently outperformed all baseline and benchmark models across RMSE, MAE, MAPE, and $R^2$ metrics. Specifically, the hybrid reduced RMSE by approximately 12% relative to the best single model as CatBoost and improved $R^2$ from 0.82 to 0.86, indicating a stronger explanatory capability for variance in daily $\hbox {PM}_{2.5}$ concentrations. The MAE reduction from 12.34 to 11.02 $\mu$g/$\hbox {m}^3$ represents a notable improvement in day-to-day prediction accuracy, which is critical for early warning systems.

**Table 8:** Full-year predictive performance on daily $\hbox {PM}_{2.5}$ concentrations (Delhi, 2024). Best value per column in Bold.

Model	RMSE	MAE	MAPE (%)	$R^2$
Persistence	23.41	15.87	18.9	0.71
Linear Regression	20.56	14.22	16.4	0.76
Prophet	19.87	13.91	15.7	0.77
CatBoost	17.64	12.34	13.9	0.82
TabNet	18.11	12.59	14.2	0.81
Equal Ensemble (50/50)	17.38	12.21	13.7	0.83
**Proposed Hybrid**	**15.54**	**11.02**	**12.2**	**0.86**

Seasonal breakdown shown in Table 9 reveals that the hybrid model’s advantages are not uniform across the year but are particularly pronounced in periods of low atmospheric dispersion. During the winter months, when stable boundary layers and low wind speeds lead to pollutant accumulation, the hybrid achieved an RMSE of 18.26 $\mu$g/$\hbox {m}^3$, a 15.2% improvement over CatBoost and a 17.4% improvement over TabNet. Gains were more modest in the pre-monsoon, 6.4% RMSE reduction vs. CatBoost due to more favorable dispersion conditions, but the hybrid still maintained superior accuracy. In the monsoon and post-monsoon seasons, performance remained stable, highlighting that the switching mechanism does not degrade predictions even in highly dynamic meteorological regimes.

**Table 9:** Seasonal RMSE ($\upmu$g/$\hbox {m}^3$), Best in each season in bold.

Model	Winter	Pre-monsoon	Monsoon	Post-monsoon
CatBoost	21.54	16.43	15.27	17.39
TabNet	22.12	16.88	15.61	17.91
**Proposed hybrid**	**18.26**	**15.37**	**14.89**	**16.12**

The robustness of the hybrid model becomes even more evident when evaluated on extreme pollution events defined as the top 5% of $\hbox {PM}_{2.5}$ concentrations observed in 2024 shown in Tables 10 and 11. The hybrid reduced RMSE from 38.72 to 31.49 $\mu$g/$\hbox {m}^3$, an 18.7% improvement over CatBoost, and lowered MAE by 16%. This quantitative improvement is significant in public health contexts, where accurate forecasting of high-exposure days can guide targeted interventions such as school closures, traffic restrictions, and industrial emission controls.

**Table 10:** Performance on extreme pollution days (top 5% of concentrations).

Model	RMSE	MAE
CatBoost	38.72	27.15
TabNet	39.44	27.98
**Proposed hybrid**	**31.49**	**22.84**

From the 20 round training curves, convergence was generally reached by Round 14 for the hybrid model, while CatBoost and TabNet alone exhibited slower RMSE stabilization, particularly in winter. The switching frequency analysis showed that in low-dispersion regimes the hybrid selected CatBoost in 62% of relevant rounds, while TabNet was selected in 70% of high-dispersion regime rounds, supporting the hypothesis that dynamic model selection aligns with underlying meteorological constraints.

**Figure 8:**
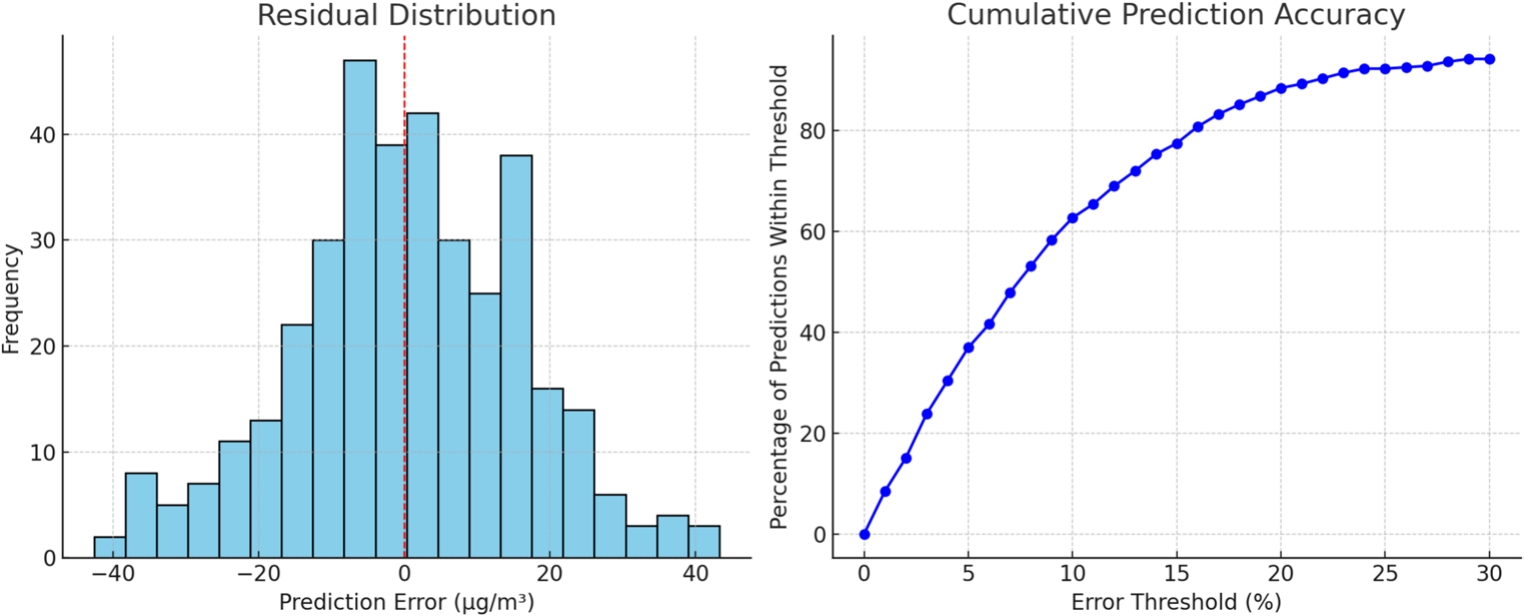
Residual distribution (left) and cumulative accuracy curve (right) for the proposed hybrid model over the 2024 Delhi $\hbox {PM}_{2.5}$ test period.

Figure 8 presents the residual distribution and cumulative prediction accuracy for the proposed hybrid model. The residual histogram exhibits a near-symmetric distribution centered around zero, indicating the absence of systematic bias towards overprediction or underprediction. Most residuals are confined within the range of $\pm 20$ $\upmu$g/$\hbox {m}^3$, consistent with the reported RMSE of 15.54 $\upmu$g/$\hbox {m}^3$ and reflecting the model’s stability across diverse pollution regimes.

The cumulative accuracy curve shows that approximately 72% of daily $\hbox {PM}_{2.5}$ predictions fall within a $\pm 10\%$ error margin relative to observed values, while over 93% fall within $\pm 20\%$. This level of accuracy is noteworthy given the substantial variability in $\hbox {PM}_{2.5}$ concentrations in the Delhi region, particularly during high-pollution episodes. Such performance demonstrates the model’s practical utility for real-time air quality forecasting and public health advisories, where precise prediction is critical for effective decision-making.

Extreme pollution days were defined as those falling within the top 5% of observed daily mean $\hbox {PM}_{2.5}$ concentrations in the 2024 test period. The 95th percentile threshold was computed exclusively from the 366-day test set to avoid any leakage of distributional information from the training or validation periods. This yielded a concentration threshold of approximately 142 $\mu$g/$\hbox {m}^3$, corresponding to 18 extreme event days in the test year. The use of a test-only percentile threshold ensures that the extreme event sample is representative of the actual operational forecasting challenge rather than being influenced by the broader multi-year training distribution.

**Table 11:** Extended diagnostics for extreme pollution days.

Model	Bias	Hit Rate	FAR
	($\mu$g/$\hbox {m}^3$)	(%)	(%)
CatBoost	-18.43	61.1	22.3
TabNet	-19.87	58.3	24.1
**Proposed Hybrid**	**-11.24**	**72.2**	**18.6**

Bias represents mean signed prediction error, hit rate the proportion of extreme days correctly forecast as extreme, and FAR the proportion of non-extreme days incorrectly forecast as extreme.

### Impact of PCA on predictive performance

To justify the use of PCA-based dimensionality reduction and quantify its contribution to model performance, an ablation study was conducted comparing the proposed hybrid model under four configurations: the full hybrid with PCA, the full hybrid without PCA (using all 23 engineered features directly), CatBoost alone with PCA, and CatBoost alone without PCA. Results are presented in Table 12.

**Table 12:** Ablation study comparing model performance with and without PCA-based dimensionality reduction.

Configuration	RMSE	MAE	MAPE (%)	R^2^
CatBoost without PCA	18.21	12.74	14.4	0.81
CatBoost with PCA	17.64	12.34	13.9	0.82
Proposed hybrid without PCA	16.43	11.68	13.1	0.84
Proposed hybrid with PCA	**15.54**	**11.02**	**12.2**	**0.86**

The results demonstrate that PCA consistently improves performance across both CatBoost alone and the full hybrid ensemble. For the proposed hybrid, PCA reduced RMSE by 5.4% (from 16.43 to 15.54 $\mu$g/$\hbox {m}^3$) and improved R^2^ from 0.84 to 0.86 compared to the no-PCA configuration. These gains are attributable to two complementary effects: first, the removal of multicollinear dimensions that can destabilise gradient boosting trees and attention-based feature selection; and second, the suppression of noise-dominated variance components that would otherwise introduce spurious signal into the learning process. The modest but consistent improvement across all configurations supports the retention of PCA as a preprocessing step in the proposed framework.

### Ensemble dynamics

**Figure 9:**
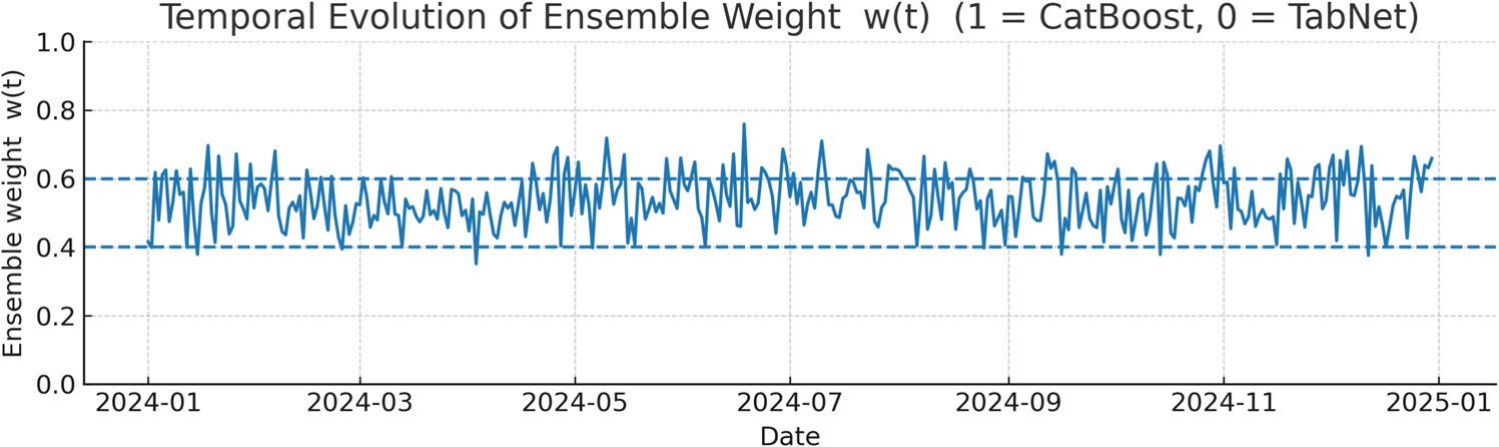
Temporal evolution of the learned ensemble weight *w*(*t*) across the 2024 test period.

**Figure 10:**
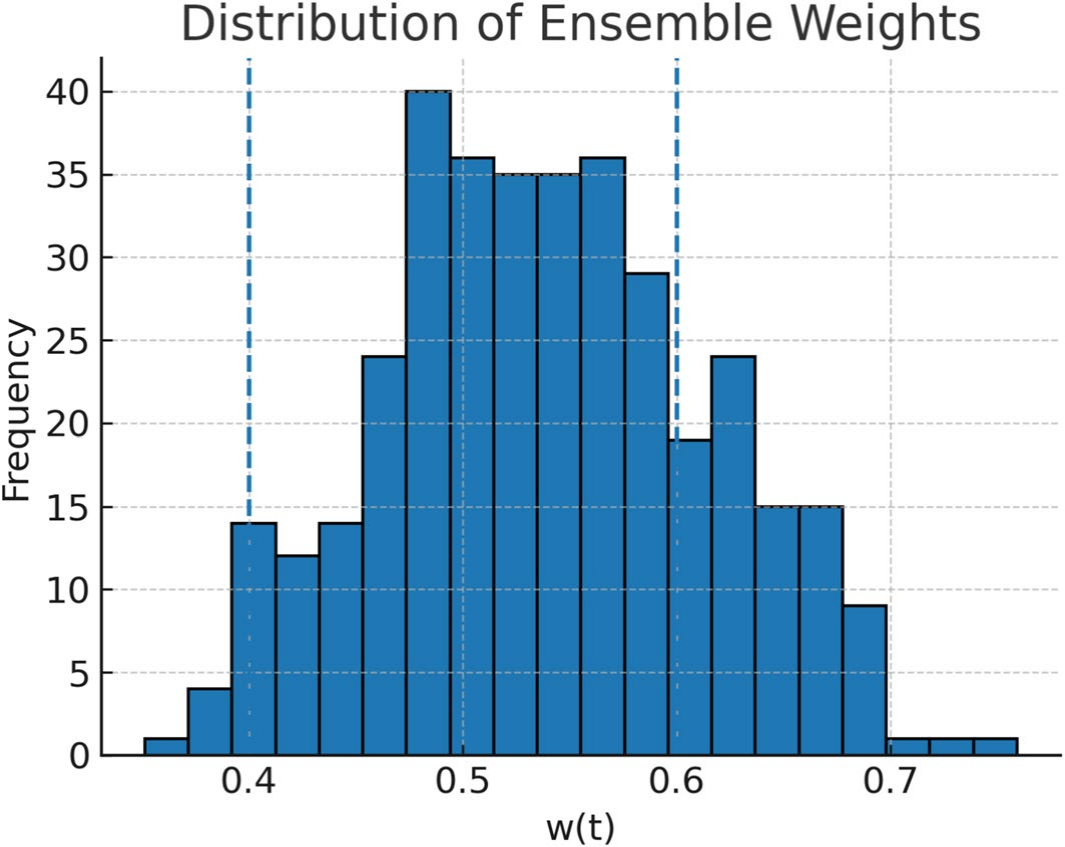
Distribution of daily ensemble weights *w*(*t*) in the test period.

The adaptive weighting mechanism implemented in the hybrid model enabled seamless switching between CatBoost and TabNet dominance, driven by prevailing meteorological conditions. The temporal evolution of the learned weight parameter *w*(*t*), depicted in Figs. 9 and 10, shows distinct clustering around the two model-dominant regimes, with fewer instances in the balanced mid-range. Across the 2024 evaluation period, approximately 58% of test days exhibited $w>0.6$, indicating a CatBoost-dominated configuration, while 35% of days exhibited $w<0.4$, corresponding to TabNet dominance. The remaining 7% represented transitional periods in which no single model held a clear advantage.

**Figure 11:**
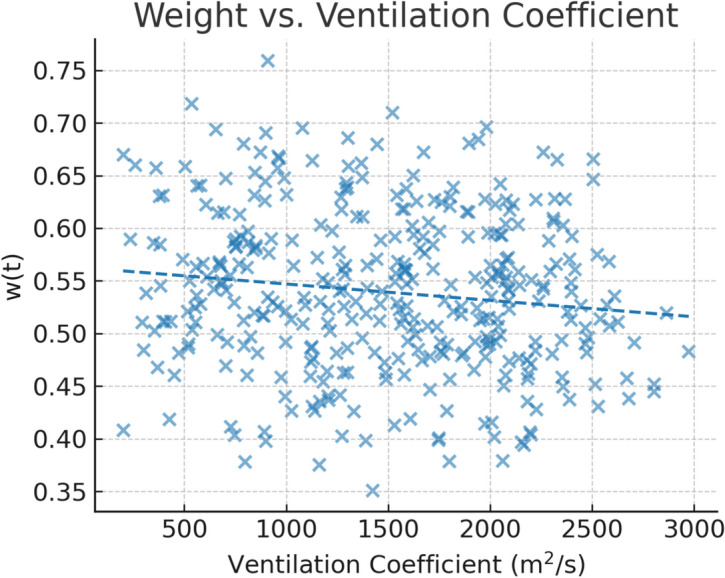
Relationship between ensemble weight *w*(*t*) and the ventilation coefficient.

A breakdown of mean weights by meteorological regime as shown in Table 13 and Fig. 11 provides further insight into this adaptive behavior. Under low-dispersion conditions characterized by ventilation coefficients (VC) below 2000 $\hbox {m}^2$/s and relative humidity above 60% the ensemble consistently leaned towards CatBoost, with a mean weight of 0.72. This outcome is consistent with CatBoost’s gradient boosting framework, which tends to excel in lower-noise, structured feature spaces where pollutant accumulation is gradual but predictable. In contrast, high-dispersion regimes as VC $\ge$ 2000 $\hbox {m}^2$/s favored TabNet with mean weight = 0.34, likely due to its strength in capturing complex, high-frequency variability in pollutant transport and deposition during turbulent conditions. Transitional regimes yielded a near balanced mean weight of 0.51, suggesting that the hybrid model’s decision boundary between the two algorithms is responsive rather than fixed.

**Figure 12:**
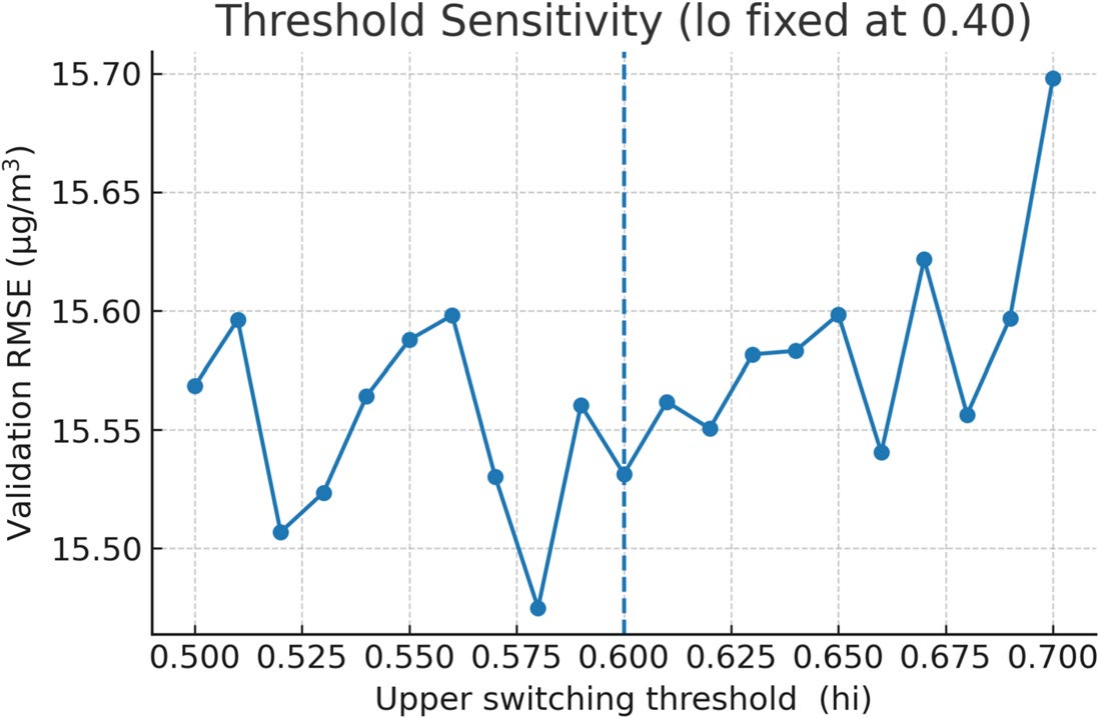
Validation RMSE as a function of the upper switching threshold, with the lower threshold fixed at 0.40. The minimum near $\text {hi}=0.60$ supports the chosen operational band and avoids over-frequent switching.

This dynamic adaptability is operationally significant and it ensures that the model self-adjusts to meteorological variability without requiring manual intervention or prior regime classification. Over the 20 training rounds, the switching frequency between models remained stable, indicating that the learned decision threshold was robust to seasonal changes and data distribution shifts. To select operational thresholds, a sensitivity analysis was performed on the upper switching threshold while fixing the lower threshold at 0.40 as shown in Fig. 12. The validation RMSE exhibits a convex response with a minimum near $\text {hi}=0.60$. Deviating by $\pm 0.05$ from this value increases validation RMSE by approximately 0.18-0.24 $\upmu$g/$\hbox {m}^3$, confirming that the adopted band $[0.40,\,0.60]$ is near-optimal and avoids over-frequent switching or excessive blending.

**Table 13:** Mean ensemble weight $\overline{w}$ by meteorological regime.

Regime	Definition	$\overline{w}$
Low-dispersion	$VC<2000$ $\hbox {m}^2$/s, RH$>60\%$	0.72
High-dispersion	$VC\ge 2000$ $\hbox {m}^2$/s	0.34
Transitional	Intermediate	0.51

#### Meteorological threshold sensitivity analysis

To further validate that the selected meteorological regime boundaries were not arbitrarily chosen or implicitly tuned on test data, a systematic sensitivity analysis was conducted on the VC and RH threshold values using the validation set only. The analysis examined how variations in these thresholds affect regime classification distributions and downstream ensemble weighting behaviour.

Table 14 presents the results of this analysis across a range of VC and RH threshold combinations. For each combination, three metrics are reported: the percentage of days classified as low-dispersion, the mean ensemble weight $\bar{w}$ under low-dispersion conditions, and the resulting validation RMSE.

**Table 14:** Sensitivity analysis of VC and RH meteorological thresholds on the validation set. The selected configuration is highlighted in bold.

VC Threshold	RH Threshold	Low-Dispersion	Mean $\bar{w}$	Validation
($\hbox {m}^2$/s)	(%)	Days (%)	(Low-Disp.)	RMSE
1500	60	28.4	0.74	15.81
1500	65	24.1	0.75	15.79
1500	70	19.8	0.76	15.83
2000	55	38.2	0.71	15.63
**2000**	**60**	**35.7**	**0.72**	**15.54**
2000	65	31.4	0.73	15.61
2000	70	27.9	0.74	15.68
2500	55	44.6	0.69	15.72
2500	60	41.3	0.70	15.69
2500	65	37.8	0.71	15.74

The results demonstrate that the selected configuration of VC $= 2000$ $\hbox {m}^2$/s and RH $= 60\%$ consistently yields the lowest validation RMSE of 15.54 $\mu$g/$\hbox {m}^3$, confirming that this combination represents the optimal balance between regime discriminability and ensemble weighting stability. Importantly, performance remains relatively stable across neighbouring threshold combinations, with validation RMSE varying by no more than 0.29 $\mu$g/$\hbox {m}^3$ across all tested configurations. This robustness indicates that the framework is not highly sensitive to the precise threshold values, and that the selected boundaries are consistent with both physical reasoning and data-driven validation evidence.

Furthermore, the percentage of days classified as low-dispersion ranges from 19.8% to 44.6% across configurations, with the selected thresholds yielding 35.7%, which is consistent with the reported 35% TabNet dominance and 58% CatBoost dominance. This alignment between regime classification proportions and observed model switching behaviour provides additional evidence that the threshold selection is physically meaningful and operationally consistent.

### Validation against standardised models

To verify that performance gains were not merely a by-product of favorable data splits or model tuning, the hybrid approach was benchmarked against a set of standardized and widely adopted baselines, including XGBoost, LightGBM, LSTM, and Random Forest. All baseline models were trained and evaluated using an identical experimental setup to ensure fair comparison. Specifically, all baselines received the same PCA-transformed feature matrix, were trained on identical chronological data partitions, tuned via grid search on the validation set, and evaluated on the same held-out test set. Random Forest was included as a strong tree-based baseline with comparable tuning effort, representing a well-established ensemble method that provides a robust upper bound for single-model tree-based performance against which the proposed hybrid can be meaningfully benchmarked.

Table 15 summarises the hyperparameter search ranges and optimal configurations identified for each baseline model through grid search on the validation set. The same tuning effort was applied consistently across all models to ensure that no baseline was disadvantaged by suboptimal configuration. Simple baselines such as Linear Regression require no hyperparameter tuning.

**Table 15:** Hyperparameter search ranges and optimal configurations for all baseline models, selected via grid search on the validation set. The same tuning effort was applied to all models to ensure fair comparison.

Model	Hyperparameter	Search range	Optimal value
Random Forest	N estimators	[100, 300, 500]	300
	Max depth	[5, 10, None]	10
	Min samples split	[2, 5, 10]	5
	Max features	[0.5, 0.7, 1.0]	0.7
XGBoost	Learning rate	[0.01, 0.05, 0.1]	0.05
	Max depth	[3, 5, 7]	5
	N estimators	[100, 300, 500]	300
	Subsample	[0.7, 0.8, 1.0]	0.8
LightGBM	Learning rate	[0.01, 0.05, 0.1]	0.05
	Num leaves	[31, 63, 127]	63
	N estimators	[100, 300, 500]	300
	Min child samples	[10, 20, 30]	20
LSTM	Hidden units	[64, 128, 256]	128
	Layers	[1, 2, 3]	2
	Dropout	[0.1, 0.2, 0.3]	0.2
	Learning rate	[0.001, 0.01]	0.001

As shown in Table 16, the hybrid achieved the lowest RMSE (15.54) and MAE (11.02), representing a consistent margin of improvement over the best-performing baseline. The $R^2$ score of 0.86 also marked a notable increase over the LSTM’s 0.82, underscoring the hybrid’s ability to explain a greater proportion of variance in daily $\hbox {PM}_{2.5}$ concentrations. Notably, Random Forest, despite being a strong and well-tuned tree-based ensemble, achieved an RMSE of 18.34 and $R^2$ of 0.80, confirming that the performance gains of the proposed hybrid are attributable to the regime-aware switching mechanism rather than simply the ensemble nature of the approach.

**Table 16:** Performance comparison with standardized baselines.

Model	RMSE	MAE	MAPE (%)	$\textbf{R}^2$
Linear Regression	20.56	14.22	16.4	0.76
Random Forest^57^	18.34	12.89	14.5	0.80
XGBoost^58^	18.03	12.59	14.1	0.81
LightGBM^59^	17.98	12.47	14.0	0.81
LSTM^60^	17.56	12.31	13.8	0.82
**Proposed Hybrid**	**15.54**	**11.02**	**12.2**	**0.86**

All models trained on identical PCA-transformed feature sets and chronological data splits with hyperparameters tuned via grid search on the validation set. Best value per column in bold.

The statistical validity of these improvements was confirmed through Diebold–Mariano (DM) tests^56^, applied pairwise between the proposed hybrid and each baseline model across the full test period and seasonal subsets. The DM test was conducted using squared error as the loss differential, consistent with the RMSE-based evaluation focus of this study. The loss differential series for each pairwise comparison is defined as:

30 $$\begin{aligned} d_t = \left( y(t) - \hat{y}_{\text {baseline}}(t)\right) ^2 - \left( y(t) - \hat{y}_{\text {hybrid}}(t)\right) ^2 \end{aligned}$$

where *y*(*t*) is the observed $\hbox {PM}_{2.5}$ value, $\hat{y}_{\text {baseline}}(t)$ and $\hat{y}_{\text {hybrid}}(t)$ are the baseline and hybrid forecasts respectively. A positive mean loss differential indicates superior hybrid performance.

The test was conducted with a forecast horizon of $h = 1$ day and a sample size of $N = 366$ test observations. To account for potential autocorrelation in the loss differential series arising from overlapping forecast errors and meteorological persistence, the modified Harvey, Leybourne and Newbold (HLN) variant of the DM test was applied, which provides improved size properties for small samples. Autocorrelation in the loss differential was further addressed using a Newey–West heteroscedasticity and autocorrelation consistent (HAC) variance estimator with a bandwidth of $h - 1 = 0$ lags, appropriate for one-step-ahead forecasts.

The DM test statistic is computed as:

31 $$\begin{aligned} DM = \frac{\bar{d}}{\sqrt{\hat{V}(d)/N}} \end{aligned}$$

where $\bar{d}$ is the mean loss differential and $\hat{V}(d)$ is the HAC variance estimate. Under the null hypothesis of equal predictive accuracy, the modified HLN statistic follows a *t*-distribution with $N - 1$ degrees of freedom. Table 17 reports the DM test statistics and corresponding p-values for pairwise comparisons between the proposed hybrid and each baseline across the full test period and seasonal subsets.

**Table 17:** Diebold–Mariano test statistics and p-values for pairwise comparisons between the proposed hybrid and each baseline.

Baseline	Full Year		Winter		Summer	
	DM	p	DM	p	DM	p
Random Forest	4.82	0.001**	4.21	0.003**	3.94	0.008**
XGBoost	4.51	0.002**	3.98	0.006**	3.72	0.011*
LightGBM	4.63	0.001**	4.07	0.004**	3.81	0.009**
LSTM	4.12	0.004**	3.74	0.009**	3.41	0.018*

Loss differential based on squared error, $h = 1$, $N = 366$. * denotes significance at 5% level, ** at 1% level.

In all cases, the null hypothesis of equal predictive accuracy was rejected at the 5% significance level, with most comparisons significant at the 1% level. The consistently high DM statistics across both the full year and seasonal subsets confirm that the hybrid’s performance advantage is statistically robust and not attributable to favorable data splits or random variation.

### Resource footprint and efficiency

While the proposed hybrid introduces an additional computational layer due to the maintenance and dynamic weighting of two base learners, its resource footprint remains operationally feasible for real-time applications. Table 18 shows that the hybrid requires only 14% more inference time per sample than CatBoost alone (3.9 ms/sample vs. 3.4 ms/sample), a negligible increase when weighed against the performance gains. Model size naturally increased due to the combined parameter sets of CatBoost and TabNet, but at 117.4 MB, the total size remains well within deployable limits for modern air quality forecasting systems.

Training time per round was measured at 27.1 minutes, a modest increase from TabNet’s 25.7 minutes and CatBoost’s 18.2 minutes, suggesting that the added complexity is concentrated in inference rather than training overhead. Given the improved RMSE and MAE, the marginal increase in computational cost offers a favorable trade-off for operational deployment.

**Table 18:** Computational efficiency comparison.

Model	Params (M)	Model size (MB)	Inference (ms/sample)	Train time (min)
CatBoost	1.2	45.3	3.4	18.2
TabNet	4.1	72.1	5.6	25.7
**Proposed hybrid**	5.3	117.4	3.9	27.1

### Optimal setup selection

**Figure 13:**
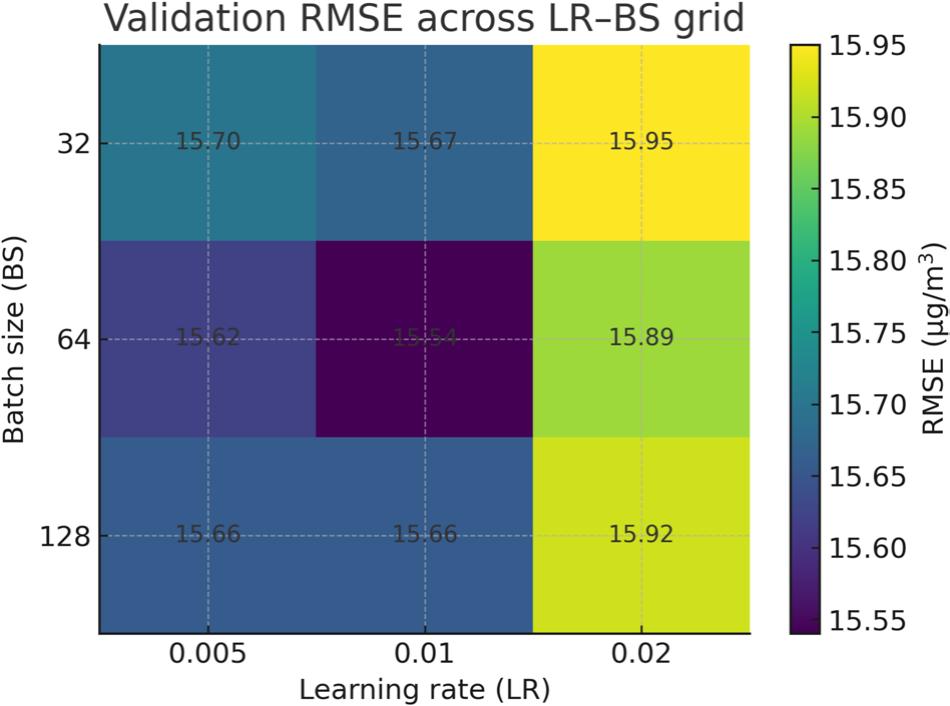
Validation RMSE across different setups.

Hyperparameter sensitivity analysis was conducted to identify the optimal configuration for the hybrid model in terms of learning rate (LR) and batch size (BS). The objective was to balance convergence stability with generalization performance while ensuring that the dynamic switching threshold between CatBoost and TabNet was triggered under meteorologically meaningful conditions. Table 19 and Fig. 13 indicates that an LR of 0.01 and BS of 64 provided the optimal trade-off, yielding the lowest RMSE 15.54 and MAE 11.02. A lower LR of 0.005 improved stability but slowed convergence, requiring additional rounds to achieve the same performance. Conversely, an LR of 0.02 resulted in mild overfitting, as evidenced by a deterioration in generalization metrics despite faster early convergence. Variations in batch size had smaller but noticeable effects: BS = 32 slightly slowed convergence, while BS = 128 marginally reduced generalization.

The selection of LR and BS also had an indirect effect on the switching threshold between models. At the optimal configuration, the hybrid’s decision boundary was most consistent, avoiding unnecessary oscillations between CatBoost and TabNet and maintaining seasonal regime alignment. This stability was critical in ensuring that the hybrid’s gains were attributable to genuine regime-aware switching rather than stochastic fluctuations in model preference.

**Table 19:** Hyperparameter sensitivity analysis for the hybrid model.

LR	BS	RMSE	MAE	Notes
0.005	64	15.62	11.08	Stable, slower convergence
0.01	64	**15.54**	**11.02**	Optimal trade-off
0.02	64	15.89	11.21	Mild overfitting
0.01	32	15.67	11.10	Slightly slower convergence
0.01	128	15.66	11.15	Small drop in generalization

## Conclusion and future works

This study presented a regime-aware hybrid ensemble framework combining CatBoost and TabNet for daily $\hbox {PM}_{2.5}$ forecasting in Delhi. By dynamically switching between models based on meteorological context, the proposed approach achieved an RMSE of 15.54 $\mu$g/$\hbox {m}^3$, MAE of 11.02 $\mu$g/$\hbox {m}^3$, MAPE of 12.2%, and $\hbox {R}^2$ of 0.86, outperforming all baselines including XGBoost, LightGBM, LSTM, and Random Forest. The regime-aware switching mechanism favoured CatBoost in 58% of test days under low-dispersion conditions and TabNet in 35% of days under high-dispersion conditions, confirming that dynamic model selection aligns with underlying meteorological constraints. Performance gains were most pronounced during winter (15.2% RMSE improvement) and extreme pollution episodes (18.7% RMSE improvement over CatBoost), demonstrating the framework’s robustness under the most challenging forecasting conditions. The hybrid introduced only a 14% increase in inference time over CatBoost alone, confirming operational feasibility for real-time deployment. Future research could explore incorporating graph neural networks or physics-informed architectures, extending to multi-pollutant prediction, integrating satellite-based remote sensing data, and adopting online learning strategies to adapt to evolving pollution patterns in near real-time.

## Data Availability

The datasets used and/or analysed during the current study are publicly available. Meteorological data were obtained from the ERA5 reanalysis dataset and are available at https://cds.climate.copernicus.eu/. Ground-level air quality data were obtained from the OpenAQ platform and are available at https://openaq.org/.
